# Sleep and Safety Improve Physicians’ Psychological Functioning at Work During Covid-19 Epidemic

**DOI:** 10.3389/fpsyg.2020.569324

**Published:** 2021-02-15

**Authors:** Nina Zupancic, Valentin Bucik, Alojz Ihan, Leja Dolenc-Groselj

**Affiliations:** ^1^Department of Psychology, Faculty of Arts, University of Ljubljana, Ljubljana, Slovenia; ^2^Institute of Clinical Neurophysiology, University Medical Centre, Ljubljana, Slovenia; ^3^Institute of Microbiology and Immunology, Faculty of Medicine, University of Ljubljana, Ljubljana, Slovenia; ^4^Department of Neurology, Faculty of Medicine, University of Ljubljana, Ljubljana, Slovenia

**Keywords:** sleep, safety, physicians, COVID-19, self-regulation, medical errors, compromised safety, psychological functioning at work

## Abstract

**Purpose:**

The COVID-19 pandemic caused a massive healthcare crisis. To investigate what makes healthcare system resilient and physicians better at coping during a crisis situation, our study investigated the role risk exposure, such as working at COVID-19 entry points, sleep, and perceived work safety played in reducing negative psychological functioning at work, as well as their effects on adverse and potentially fatal incidences of compromised safety and medical errors.

**Methods:**

Our study included a representative sample of 1,189 physicians, from all 12 Slovenian regions and all medical occupations, as registered by the Medical Chamber of Slovenia. For the purposes of this study, a Questionnaire of Sleep and Psychological Functioning at Work was developed in the form of an online retrospective self-report. Additionally, our study included items assessing physicians perceived work safety and frequency of negative outcomes (compromised safety and medical errors) during the first month of the Covid-19 epidemic.

**Results:**

Physicians working at COVID-19 entry points were more likely to experience night awakening, slept less than 5 h per night, experience nightmares, and had lower levels of psychological functioning in comparison to other physicians. Both hypothesized models showed adequate fit. A higher score on the sleep scale (sleep quantity, sleep quality, and shorter sleep latency) has been shown to predict lower levels of negative psychological functioning at work and, indirectly, reduced incidences of compromised safety and medical errors. Contrary to our expectations, no significant direct effect of sleep on compromised safety and medical errors was found. When perceived work safety was added into the model, the model showed improved fit, with perceived work safety predicting better sleep, less negative psychological functioning at work, and less compromised safety.

**Conclusion:**

Sleep and safety both play an important role in reducing negative psychological functioning at work and, by doing so, decreasing the negative and potentially fatal incidents during the pandemic, such as compromised safety and medical errors. Further, research is needed to see how medical guidelines can be updated to ensure physicians sleep and that their safety is protected.

## Introduction

The COVID-19 pandemic caused a massive healthcare crisis that many affected countries attempted to address with the national first-point-of-contact strategy for possible COVID-19 cases, as recommended by World Health Organisation (2020). This approach protects healthcare professionals in primary care centers and hospitals, as well as individuals who perform other services in these institutions. In Slovenia patients with signs of acute respiratory infection with or without fever were directed to COVID-19 entry points (Ministrstvo za zdravje-Republika Slovenija, 2020). For outpatients that did not necessarily require hospital care, COVID-19 entry points were in healthcare centers across the country, where primary level physicians performed the testing for COVID-19 infection. For inpatients that required hospital treatment, entry points were located within emergency medical care units. Medical Chamber of Slovenia was concerned that the establishment of COVID-19 entry points within emergency medical care units or at primary health care centers further increased the risk of infection spread onto patients without infection that needed to wait up to 3 h to receive their test results. They believed that the COVID-19 entry points should be established outside of the premises of healthcare facilities by National Institute for Public Health (NIJZ) and handled exclusively by epidemiologists (Čebašek-Travnik et al., 2020). Furthermore, establishment at the primary level hospital has increased concerns due to the lack of clear guidelines, difficulties in establishment of appropriate spaces, limited access to protective gear, and most importantly, it provided additional responsibilities in the diagnosis of COVID-19 to general practitioners (Klim, n.d.), which were already severely understaffed and overwhelmed prior to the epidemic (Republika Slovenija Državni Zbor, 2019; Klim, n.d.). By the end of July 2020, 17% of all infections with COVID-19 in Slovenia were diagnosed among healthcare workers or workers in other care facilities (NIJZ, 2020). This has shown to be a major contributing factor in some of the regions with the highest infection rate, such as Šmarje pri Jelšah, Metlika, and Ljutomer, where the infections among healthcare workers or long-term care workers have shown to be the important contributors toward the spread of the infection (Motoh, 2020). Our study aims to understand how perceived work safety and exposure to risk, such as working at COVID-19 entry points, could have impacted physician sleep and psychological functioning at work and whether sleep and safety could have worked as protective factors in ensuring resilient healthcare system by decreasing the likelihood of compromised safety and medical errors.

Understanding the sleep of physicians in relation to the COVID-19 response is important as: (1) Sleep deprivation increases the likelihood and subsequent adverse outcomes of infection (Patel et al., 2011; Prather and Leung, 2016). (2) Sleep loss decreases cognitive and emotional functioning of physicians (Zohar et al., 2005), increasing the likelihood of adverse outcomes, such as medical errors and compromised safety (Barger et al., 2006; Lockley et al., 2007; Brossoit et al., 2019). Short sleep of less than 7 h (Watson et al., 2015) limits the amount of restoration one receives during the night, while low sleep quality, referring to insomnia symptoms, such as difficulties in falling asleep, maintaining sleep, or frequency of waking in the middle of the night, can disrupt recovery processes (Scott and Judge, 2006; Harvey et al., 2008; Barnes, 2012; Litwiller et al., 2017; Medic et al., 2017). Additionally, some authors propose that daytime sleepiness can be considered as an indicator of insufficient sleep (Johns, 1992; Akerstedt et al., 2014).

Research from Wuhan, China, during the first 2 months of the COVID-19 outbreak showed that sleep quality played an important role in self-efficacy and anxiety levels among healthcare professionals working with COVID-19-infected patients (Xiao et al., 2020). Sleep affects one’s cognitive, emotional, and behavioral self-regulation and by doing so decreases the ability of individuals to perform well at work. Self-regulation can be defined as a process through which individuals navigate and modify goal-directed activities by controlling thoughts, attention, affect, and behavior (Karoly, 1993; Baumesiter et al., 2011; Barnes, 2012; Brossoit et al., 2019). Sleep deprivation decreases working memory functioning and thereby significantly increases the time needed to complete tasks, the likelihood of attention mishaps, and ones’ susceptibility to be distracted by emotional stimuli (Alhola and Polo-Kantola, 2007; Walker, 2009; Barnes, 2012). Decrease in cognitive performance may further be amplified, if sleep restriction lasts for a longer period of time (Van Dongen et al., 2003). Low task completion due to the problems an individual encounters with self-regulation can furthermore increase the likelihood of experiencing negative affect, as research on nurses has shown that daily task completion has been linked to an increase in positive affect and decrease in negative affect (Gabriel et al., 2011). Neurological studies have shown that sleep participates in habituation processes and reduces aversive reactions to stressful stimuli (Deliens et al., 2014). This may be especially crucial during the COVID-19 pandemic as healthcare workers are at an elevated risk of experiencing emotional distress (Rangachari and Woods, 2020). Resilience can buffer the effects of negative affectivity resulting from low task completion at work (Gabriel et al., 2011), with Artuch-Garde et al. (2017) study showing that there is a strong overlap between constructs of self-regulation and resilience. Resilience is characterized as a dynamic and flexible process of adaptation to changes, which can act as a buffer to stress and is a protective factor against psychological distress and mental health disorders (Montero-Marin et al., 2015; Arrogante and Aparicio-Zaldivar, 2017). Similarly to self-regulation, an individual’s resilience has been linked to higher quantity and quality sleep (Germain and Dretsch, 2016; Sher, 2020). A resilient healthcare system is crucial for fighting infectious diseases (Nuzzo et al., 2019), with the new definition of safety in healthcare settings as proposed by WHO, emphasizing resilience abilities and ability to respond to changing environment in order to protect safety (Sujan et al., 2019), which, however, does not occur without the healthcare professionals’ ability to remain resilient (Jensen et al., 2008; McCann et al., 2013).

**FIGURE 1:**
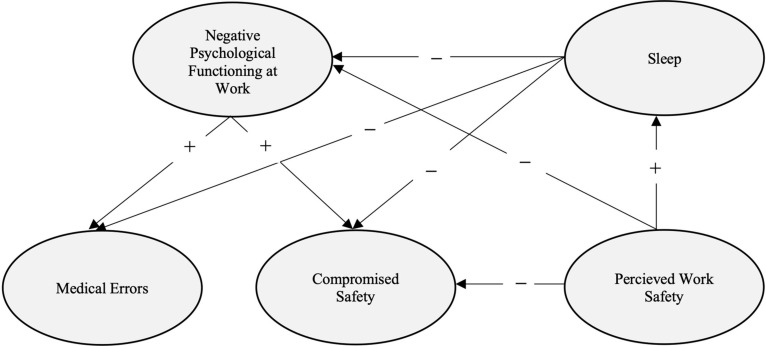
Hypothesized model of the effects of sleep and perceived work safety on psychological functioning at work and its relationship with compromised safety and medical errors.

Emerging research shows that healthcare workers, working directly with COVID-19-infected patients, were more likely to develop symptoms of depression, anxiety, and insomnia (Huang and Zhao, 2020; Pappa et al., 2020; Zhang et al., 2020). Rangachari and Woods (2020) argue that decreased psychological safety and emotional distress felt by healthcare workers during COVID-19, further contributed toward restricting organizational resilience and adversely impacted patients’ safety. Nevertheless, very little research explores the effects this might have had on physician’s work. To investigate the role sleep and perceived work safety had on physician’s work, we tested the hypothetical model as shown in Figure 1. The model was based on the following assumptions. Sleep will decrease negative psychological functioning at work, incidences of compromised safety, and medical errors (*Hypothesis 1*). Negative psychological functioning at work will increase the incidences of compromised safety and medical errors (*Hypothesis 2*). Perceived work safety will be linked to better sleep, less negative psychological functioning, and lower levels of compromised safety (*Hypothesis 3*).

## Materials and Methods

### Participants and Procedure

On March 25, the Medical Chamber of Slovenia, in this case acting as the intermediary, sent the Questionnaire of Sleep and Psychological Functioning at Work to 9,727 registered physicians, of which 1,193 responded (12% response rate). The study was preregistered at the Department of Psychology at the University of Ljubljana, and the questionnaire was uploaded on 1ka.si (an online Slovenian platform used for research purposes). On the front page, physicians were informed about the purpose of the study, the right to withdraw, usage of the information, while anonymity of their responses was ensured. The study was conducted as a part of a larger survey designed to develop a measure assessing sleep and psychological functioning at work for physicians, including additional questions used in order to provide recommendations on how to improve emergency response to COVID-19. The questionnaire was presented on seven different pages with an average survey time of 10 min. After the participants submitted their responses, they were unable to return and change their submission. The study was conducted in accordance with the Declaration of Helsinki and was approved by the Executive committee of the Medical Chamber of Slovenia. Before the survey was launched, a pilot study was conducted on a small sample of physicians (*n* = 21). Based on the initial analysis and feedback provided by participants, the measures proposed in the questionnaire were adapted and improved. The items included in the survey were derived from theories, as well as following examples of pre-existing and pre-established measures assessing sleep, self-regulation, resilience, emotions, safety, and medical errors.

### Measures

#### Demographics, COVID-19, and Work-Related Information

The questionnaire included questions regarding gender, age, illnesses, the nature of work (specialization, levels of hospital care, night shift work, absence from work, and region of work) as well as COVID-19-related characteristics (working at a COVID-19 entry point and exposure to COVID-19).

#### Sleep

The scale assessing sleep was constructed based on theory and following examples of pre-existing and validated measures of sleep. To assess the fit of the model, exploratory and confirmatory factor analysis was conducted, indicating three-factor (shorter-version) and four-factor structure (longer-version). Based upon psychometric analysis, items referring to sleep apnea, subjective sleep evaluation, and medicine taking were excluded from the measure. For the purpose of this study, a shorter version including nine items was used with three-dimensional factor structure (sleep quality, quantity, and latency). Scores on sleep scale are calculated as a sum of all dimensions (sleep quantity, quality, and latency) with the lowest score 0 and the highest score 30. Physicians reported the occurrence of sleeping problems on a four-point Likert scale (*3*–*never, 2–less than once a week, 1–once or twice a week, 0–three or more times a week*). Items referring to *sleep quantity* included sleep duration on workdays and non-workdays (0—< 6 h, 1—6–7 h, 2—7–8 h, 3—8–9 h, 4—9–10 h, 5— >10 h) and the occurrence of reduced sleep (<5 h sleep). Items referring to *sleep quality* included questions on the occurrence of insomnia symptoms and nightmares. *Sleep latency* included items of average sleep latency (0—*less than 30 min, 1.5—from 30 to 60 min, 3—more than 60 min*) and the occurrence of delayed sleep latency (> 30 min). Given that the items referring to frequency of reduced sleep (<5 h) and average sleep latency were scored on different continuums to the dimension of sleep quantity and quality, the scoring of the items was transformed to allow equal weights among indicators. The total score of sleep (0–30) is calculated as a sum of all the total scores on dimensions of sleep quantity (0–15), sleep latency (0–6), and sleep quality (0–16) (Appendix 1).

#### Psychological Functioning at Work

A scale was developed to assess potential self-regulatory failures, experience of negative emotions, and resilience at work. In our scale development, we followed example similar measures assessing reduced cognitive and emotional regulation at work, negative affectivity, where we have specifically added items that refer to emotions that physicians could have experienced during crisis and could have impacted their work. Finally, we have added items assessing resilience based on previous measures and literature on healthcare workers (Jensen et al., 2008; McCann et al., 2013). Items were scored on a five-point Likert scale (1—*never*, 2—*rarely*, 3—*sometimes*, 4—*often*, 5—*very often*). The measure consists of seven items assessing self-regulatory failures (decision making, memory problems, attention deficits, emotional regulation in interaction, empathy), five items assessing negative affectivity (feelings of powerlessness, fear, anger, sadness and concern), and five items referring to resilience (adaptation, coping, positivity, feeling strong and capable, energy, self-efficacy). To allow for the comparability of different dimensions, we have averaged the score of specific dimensions and total score. The scale can be scored both on the negative as on the positive end of the continuum (Appendix 2).

#### Compromised Safety and Medical Errors

Items referring to incidences of compromised safety and medical errors in the first month of COVID-19 epidemic (“*In the past month, how often on average did.”*) were measured on a 4-point scale (1—*never*, 2—*less than or once a week*, 3—*two or three times a week*, 4—*more than three times a week*).

#### Perceived Work Safety

*Three items measured participants’ level of agreement (*“To what extent do you agree, with each of the statements that it was true for you or your work environment in the past month…”*) on statements referring to perceived work safety (*…the safety of employees was well taken care of, …you were provided with protective gear in sufficient quantities, …you felt safe and protected*) based on a five-point Likert scale (1—*completely disagree, *2—*disagree, *3—*neither agree nor disagree, *4—*agree, *5—*completely agree*).*

#### Sleepiness

The Slovenian version of the Epworth Sleepiness Scale (ESS; Johns, 1992) was used to assess the usual level of daytime sleepiness. The ESS is a widely used and validated tool, where respondents report their likelihood of falling asleep in different daily situations on a four-point scale (0*—would never doze*, 1*—slight chance of dozing*, 2*—moderate chance of dozing*, 3*—high chance of dozing*). Final scores are summed, and higher score indicates greater sleepiness, with score above 10 indicating excessive daytime sleepiness (Spira et al., 2011).

### Statistical Analysis

The data was analyzed using SPSS and R statistical software. First, we conducted reliability analysis for items assessing sleep and psychological functioning at work with Cronbach alpha and McDonald’s omega indexes. Then we performed exploratory factor analysis in SPSS to identify optimal factor structure. To establish construct validity, we performed confirmatory factor analysis using Lavaan package in R. After testing for assumptions, multivariate linear regression analysis was used to assess the predictor power of items that were finally added in the model. We used structural equation modeling in the package Lavaan referring to Robust Maximum Likelihood to assess the fit of the models, as some items showed significant deviations from normality. In our evaluation of the model, we followed the guidelines proposed by Marsh et al. (2005) and the European Journal of Psychological Assessment (Schweizer, 2010). Binary logistic regression analysis was performed in order to investigate the potential differences among physicians working at COVID-19 entry points and others.

## Results

Our study included a representative sample of physicians working in all 12 geographical regions of Slovenia. The number of physicians that participated in the study was the highest for the two regions with the largest population size (Central Slovenia and the Drava region). Surveys, 1,019, were completed; nevertheless, after the initial analysis, four participants were excluded from the analysis, as they have not met the criteria of being employed either full-time or part-time at the time of the study (the total number of included participants was *n* = 1,189). The majority of physicians included in the sample was employed full-time (994, 92.55%), and 7.45% (80) of the subjects reported working part-time or being in a different contractual relationship (missing = 115). The sample predominantly consisted of female participants (787, 73%) and a smaller proportion of male participants (287, 27%), which is in line with the demographics of Slovenian physicians, as the Eurostat (2019) report suggests that approximately 60% of physicians in Slovenia are women. The sample included physicians working in all 54 specializations listed by the Medical Chamber of Slovenia, with the largest sample of physicians in general practice (224, 20.86%), dental medicine (130, 12.1%), pediatrics (87, 8.1%), intervention medicine (68, 6.33%), gynecology and obstetrics (68, 6.33%), neurology (41, 3.81%) and anesthesiology, rheumatology, and perioperative intensive medicine (39, 3.63%). Three hundred five (28.4%) physicians were diagnosed with chronic illness, 18 (1.68%) with mental illness, and 8 (0.75%) physicians reported having been diagnosed with a sleep disorder. The majority of participants reported they were in a relationship or married with children (692, 64.43%) or in a relationship without children (205, 19.09%); a smaller proportion of participants reported they were single, divorced, or widowed without children (99, 9.22%). The average age of participants was 45.6 years (*SD* = 11.56), with the youngest participant being 25 years of age and the oldest 84 years of age. As shown in Table 1, the sample was evenly distributed across all age groups. Three participants included in the sample reported they were infected, while 153 (12.97%) participants reported they were in close contact with someone who was infected, and 210 (17.78%) physicians reported that their co-workers were infected with COVID-19 (Table 1).

**TABLE 1:** Demographic and work-related characteristics of physicians (*n* = 1,189).

	*F*	%
**Gender** (*n* = 1,074)		

Female	787	73.28
Male	287	26.72
**Age (years) (*n* = 1,074)**		

25–32	182	16.95
33–40	216	20.11
41–48	224	20.86
49–56	229	21.32
57–64	167	15.55
>65	56	5.21
**Geographical region of work (*n* = 1,074)**		

Mura region	39	3.63
Drava region	171	15.92
Carinthia	34	3.17
Savinja region	93	8.66
Central Sava	15	1.4
Lower Sava	17	1.58
Southeast Slovenia	52	4.84
Central Slovenia	430	40.04
Upper Carniola	83	7.73
Littoral-inner Carniola	28	2.6
Gorizia	60	5.59
Coastal Karst	52	4.84
**Family status (*n* = 1,074)**		

Single, divorced, widowed without children	99	9.22
In a relationship or married without children	205	19.09
Single, divorced, widowed with children	65	6.05
In a relationship or married with children	692	64.43
Other	13	1.21
**Levels of hospital care (*n* = 1,074)**		

Primary hospital	524	48.79
Secondary hospital	284	26.44
Tertiary hospital	266	24.77
**Working at Covid-19 entry point (*n* = 1,180)**		

Working at Covid-19	319	27.03
Not working at Covid-19	861	72.97
**Covid-19 exposure (*n* = 1,180)**		

Infected with Covid-19	3	0.25
Close contact with someone infected with Covid-19	153	12.97
Co-workers infected with Covid-19	210	17.78
**Nightshift work per month (*n* = 1,049)**		

0 days	620	59.33
1–5 days	370	35.41
6–10 days	50	4.78
11–15 days	9	0.86
**Absence from work per month (*n* = 1,049)**		

0 days	438	41.92

1–5 days	313	29.95
6–10 days	205	19.62
11–20 days	76	7.27
>20 days	13	1.24

*Number of respondents on specific items differs due to missing data.*

Table 2 shows sleep duration and sleepiness of physicians during the first month of the COVID-19 epidemic in relation to psychological functioning at work. Overall, the results show that the majority of physicians slept less than what is recommended by the American Academy of Sleep Medicine (Watson et al., 2015), i.e., 6–7 h on workdays (531, 51.5%), and the second largest group of physicians slept less than 6 h per night (299, 28.9%). On non-workdays, physicians slept longer on average: the majority of participants slept for the recommended period of 7–8 h (390, 37.83%) and 8–9 h (227, 22.02%). Nevertheless, a substantial proportion of physicians reported sleeping between 6 and 7 h per night (281, 27.16%) or less than 6 h per night (74, 7.18%) on non-workdays. The largest group of physicians fell in the category of normal sleepiness according to the Epworth Sleepiness Scale (809, 79.39%) or within mild sleepiness (154, 15.11%), with a smaller proportion of physicians having moderate (35, 3.43%) or severe sleepiness symptoms (21, 2.06%). The majority of respondents needed less than 30 min to fall asleep (722, 70.02%), the second largest group on average 30–60 (248, 24.05%), and the smallest group of physicians needed more than 60 min to fall asleep (61, 5.92%). Most physicians experienced night awakening three or more times a week on average (377, 36.08%), the second largest group of physicians two or more times a week (311, 29.76%), the third largest group less than once a week (233, 22.3%), while a small proportion of physicians (10.53%) (110) reported no incidence of night awakening during the month of the COVID-19 epidemic. On the other hand, the majority of physicians reported having no difficulties falling back asleep after nocturnal awakening during the month of the COVID-19 epidemic (313, 29.95%), or experienced such difficulties less than once a week (290, 27.75%), with 233 (22.23%) physicians experiencing such difficulties once or twice a week and 195 (18.66%) experiencing such difficulties three or more times a week. A majority of physicians reported having no nightmares in the past month (440, 42.68%) or having them less than once a week (328, 31.81%), with a smaller proportion of physicians reporting such problems once or twice a week (186, 18.04%), and three or more times a week (77, 7.47%). The largest group of physicians fell in the category of normal sleepiness according to the Epworth Sleepiness Scale (809, 79.39%) or within mild sleepiness (154, 15.11%), with a smaller proportion of physicians having moderate (35, 3.43%) or severe sleepiness symptoms (21, 2.06%).

**TABLE 2:** Descriptive statistics for sleep dimensions (sleep quality, sleep latency, and sleep quantity) and daytime sleepiness in the first month of the COVID-19 epidemic (*n* = 1,189).

Sleep quantity (*n* = 1,031)	*f*	*%*
Workdays^a^		
<6 h	299	29
6–7 h	531	51.5
7–8 h	171	16.59
8–9 h	28	2.72
9–10 h	2	0.19
>10 h	1	0.1
**Non-work days^a^**		
<6 h	74	7.18
6–7 h	281	27.16
7–8 h	390	37.83
8–9 h	227	22.02
9–10 h	41	3.98
> 10 h	18	1.75
	***M***	***SD***
	
Frequency of reduced sleep (<5 h)^c^	2.13	0.89
**Sleep latency**	***f***	**%**

Average latency^b^		
<30 min	722	70.02
30–60 min	248	24.05
>60 min	61	5.92
	***M***	***SD***
	
Frequency of30 min sleep latency^c^	1.75	1.1
**Sleep quality^c^**	***M***	***SD***

Night awakening	1.07	1.01
Early waking onset	1.33	1.09
Difficulties falling back asleep after night awakening	1.70	1.09
Nightmares	2.10	0.95
**Level of sleepiness^d^ (*n* = 1,019)**	***f***	***%***

Normal	809	79.39
Mild	154	15.11
Moderate	35	3.43
Severe	21	2.06

*Pairwise deletion was performed to treat the missing values. ^*a*^“How long (in hours) have you slept on average per night during the past month? (0—< 6 h, 1—6–7 h, 2—7–8 h, 3—8–9 h, 4—9–10 h, 5— >10 h).” ^*b*^“How much time have you needed on average in the past month to fall asleep? (0—less than 30 min, 1.5—from 30 to 60 min, 3—more than 60 min).” ^*c*^“In the past month, how often has it occurred to you on average…” (3—never, 2—less than once a week, 1—once or twice a week, 0—three or more times a week). ^*d*^Epworth sleepiness scale classification: Normal (0–10), Mild (11–14), Moderate (15–17), Severe (>18).*

Confirmatory factor analysis showed an adequate fit of the proposed hierarchical model for sleep scale with Robust Maximum Likelihood statistics χ^2^ = 125.61, *df* = 25, χ^2^/*df* = 5.02, *p* = 0.000, *CFI* = 0.96, *TLI* = 0.94, *RMSEA* = 0.07, 90% *CI* (0.05, 0.07), *p* = 0.02, *SRMR* = 0.05. The total score showed good overall reliability (α = 0.79, ω = 0.87) and adequate reliability of all three subdimensions referring to the parameters of sleep quantity (α = 0.58, ω = 0.76), quality (α = 0.76, ω = 0.78), and latency (α = 0.84, ω = 0.87). Moderate positive correlations between all three dimensions of sleep indicate good multivariate outcome (*r* = 0.29–0.46, *p* < 0.001). Further on, we investigated the fit of the model for psychological functioning at work. Exploratory factor analysis indicated potentially three-dimensional factor structure, with high eigenvalue on first factor loading indicating potentially hierarchical factor structure. The model showed adequate fit for a hierarchical structure, and to improve the model fit, six indicators on latent dimensions were allowed to co-vary. Maximum likelihood χ^2^ = 619.02, *df* = 108, *p* < 0.001, χ^2^/*df* = 5.73, *CFI* = 0.95, *TLI* = 0.93, *RMSEA* = 0.06, 90% *CI* (0.06, 0.07), *p* = 0.001, *SRMR* = 0.04. Reliability analysis of the questionnaire showed excellent reliability overall (α = 0.92, ω = 0.92) and in the specific dimensions of negative affectivity (α = 0.88, ω = 0.88), negative self-regulatory processes (α = 0.81, ω = 0.75), and resilience (α = 0.86, ω = 0.85).

The sleep total score showed small negative significant correlations with the total score of the Epworth Sleepiness Scale (*r* = −0.25, *p* < 0.001), as well as dimensions on *sleep quantity* (*r* = −0.27, *p* < 0.001), *sleep quality* (*r* = −0.25, *p* < 0.001), but with a very small, although significant, correlation with *latency* (*r* = −0.06, *p* < 0.05), indicating the validity of the measurement. Small to moderate significant positive association was found between all dimensions of sleep and the average score of psychological functioning of physicians at work (*r* = *0*.17–0.46, *p* < 0.001). Negative moderate relationship was found between sleep and self-regulatory failures (*r* = −0.34, *p* < 0.001) as well as negative affectivity (*r* = −0.41, *p* < 0.001), while resilience has shown to be positively related to sleep (*r* = 0.29, *p* < 0.001). Psychological functioning of physicians at work was negatively associated to physicians’ total score on sleepiness (*r* = *−0.25, p* < 0.001). A lower score on sleep parameters and a higher score on daytime sleepiness was positively related to the incidence of physicians’ individual compromised safety at work (*r* = −0.14, *p* < 0.001; *r* = *0*.12, *p* < 0.001, respectively); similarly, a significant negative relationship was found between psychological functioning at work and incidences of compromised safety (*r* = −0.27 to −0.29, *p* < 0.001) as well as incidences of medical errors reported (*r* = −0.12 to −0.33, *p* < 0.001). The dimension of self-regulatory failures, specifically, was positively related to more compromised safety reported (*r* = 0.25–0.29, *p* < 0.001), with a significant positive moderate correlation between self-regulatory failures and medical errors committed due to exhaustion (*r* = *0*.38, *p* < 0.001) and a small significant correlation to life-threatening medical errors (*r* = 0.12, *p* < 0.001). A significant negative relationship was found between resilience and compromised safety (*r* = −0.2 to −0.23, *p* < 0.001) and medical errors (*r* = −0.13 to −0.25, *p* < 0.001). A significant positive relationship was found between negative affectivity and compromised safety, which was higher for individual compromised safety (*r* = 0.2–0.27, *p* < 0.001), as well as for medical errors due to exhaustion (*r* = *0*.2, *p* < 0.001). Furthermore, perceived work safety at the time of the COVID-19 epidemic was significantly related to lower psychological functioning at work (*r* = −0.31, *p* < 0.001), lower compromised safety (*r* = −0.26 to −0.39, *p* < 0.001), and lower number of life-threatening medical errors (*r* = −0.11, *p* < 0.001) (Table 3).

**TABLE 3:** Means (M), Standard Deviations (SD), Alpha (α), Omega (ω), and Pearson Correlations between (sub)dimensions of sleep, psychological functioning at work, sleepiness, perceived work safety, medical errors, and compromised safety during the first month of the COVID-19 epidemic (*n* = 1,189).

		*M*	*SD*	α	ω	1	2	3	4	5	6	7	8	9	10	11	12	13	14
1	**Sleep** (*n* = 1,031)	16.83	5.75	0.78	0.87	–													
2	Sleep quantity	6.42	2.53	*0.59*	*0.76*	0.71***	–												
3	Sleep quality	6.2	3.17	*0.76*	*0.78*	0.84***	0.31***	–											
4	Sleep latency^a^	4.21	1.85	*0.84*	*0.87*	0.7***	0.29***	0.46***	–										
5	**Epworth daytime sleepiness** (*n* = 1,019)	7.07	4.23	*0.9*	*0.89*	−0.25***	−0.27***	−0.21***	−0.06*	–									
6	**Perceived work safety**^b^ (*n* = 1,118)	2.83	1.11	0.9	0.89	0.16***	0.11***	0.15***	0.09**	−0.09**	–								
7	**Psychological functioning at work**^c^ (*n* = 1,189)	3.48	0.68	0.92	0.92	0.42***	0.17***	0.45***	0.3***	−0.25***	0.31***	–							
8	Self-regulatory failures	2.25	0.66	0.81	0.75	−0.34***	−0.14***	−0.37***	−0.23***	0.25***	−0.22***	−0.88***	–						
9	Resilience	3.55	0.78	0.86	0.85	0.36***	0.14***	0.39***	0.25***	−0.19***	0.3***	0.85***	−0.62***	–					
10	Negative affectivity	2.98	0.94	0.92	0.92	−0.41***	−0.17***	−0.43***	−0.3***	0.2***	−0.31***	−0.89***	0.66***	−0.65***	–				
	**Medical errors**^d^ (*n* = 1,118)			0.30	0.57														
11	… *You make a medical error due to exhaustion.*	1.33	0.55			−0.15***	−0.07*	−0.14***	−0.12***	0.1**	−0.11***	−0.33***	0.38***	−0.25***	0.22***	–	–		
12	… *You make a life-threatening medical error.*	1.04	0.2			–0.02	0.01	–0.04	0.01	–0.03	0.02	−0.12***	0.12***	−0.13***	0.07*	0.28***			
	**Compromised safety**^d^ (*n* = 1,118)			0.54	0.56														
13	… *Your actions endanger your own safety.*	1.69	0.8			−0.15***	−0.1**	−0.14***	−0.08*	0.12**	−0.31***	−0.29***	0.25***	−0.23***	0.27***	0.26***	0.14***	–	
14	… *Your actions endanger safety of other employees.*	1.23	0.49			−0.09**	–0.02	−0.12**	−0.07*	0.08**	−0.12***	−0.27***	0.29***	−0.2***	0.2***	0.39***	0.23***	0.42***	–

Pairwise deletion was performed to treat the missing values. ^*a*^High score on sleep latency dimension meant lower time to fall asleep. ^*b*^The average score was calculated for perceived work safety. Physicians rated their level of agreement on items assessing their perception of safety (1—completely disagree, 2—disagree, 3—neither agree nor disagree, 4—agree, 5—completely agree). ^*c*^The average score for psychological functioning at work was calculated and transformed on the positive continuum. “In the past month, how often did you at work…” (1—never, 2—rarely, 3—sometimes, 4—often, 5—very often). ^*d*^The average score was calculated for medical errors and compromised safety. “In the past month, how often on average did.” (1—never, 2—less then or once a week, 3—two or three times a week, 4—three or more times a week). *p < 0.05. **p < 0.01. ***p < 0.001.

**FIGURE 2:**
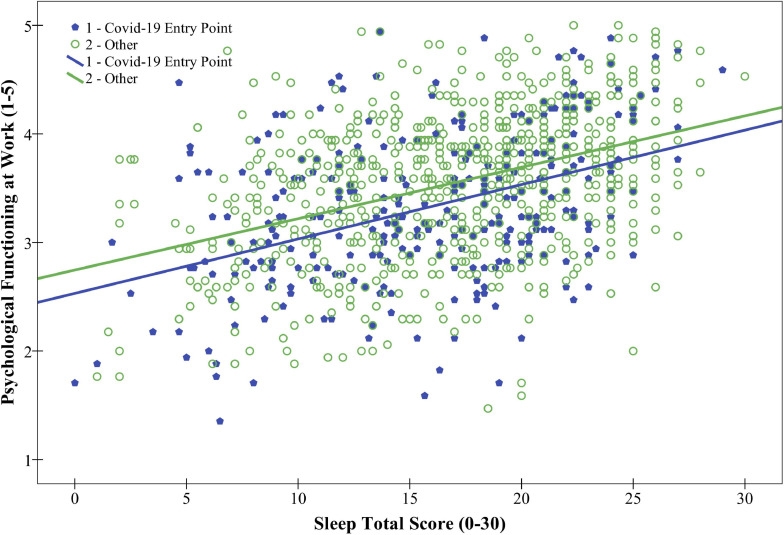
Relationship between sleep and psychological functioning at work, for groups of physicians working at COVID-19 entry points (*n* = 319) and other physicians (*n* = 861).

As can be seen in Figure 2, there is an indication of weak positive linear relationship between the total score on sleep and the average psychological functioning at work. The group of physicians who worked at a COVID-19 entry point had consistently lower scores on psychological functioning at work for each score on sleep than physicians who did not. To investigate the relationship further, we performed multiple linear regression analysis (Table 4). All predictors were significant predictors, and sleep proved to be the strongest predictor of an increase in the physicians’ psychological functioning at work (β = 0.43, *p* < 0.001, *R*^2^ = 0.18, *p* < 0.001). When the total score on sleepiness was added, the model showed a small significant improvement (Δ*R*^2^ = 0.02). Physicians that experienced more daytime sleepiness showed a significant decrease in their psychological functioning at work (β = −0.13, *p* < 0.001), while positive perception of work safety at the time of the COVID-19 epidemic increased physicians’ psychological functioning at work (β = 0.24, *p* < *0.001*) and provided improvement to the model (*R*^2^ = 0.26, Δ*R*^2^ = 0.06, *p* < 0.001).

**TABLE 4:** Multiple linear regression investigated the effects of sleep, sleepiness, perceived work safety, and working at a COVID-19 entry point on physicians’ psychological functioning at work (*n* = 1,189^a^).

		Standardized coefficients		Unstandardized coefficients					
Model		*B*	*SE*	β	*p*	*R*^2^	Δ*R^2^*	*F*	*p*
1	Sleep total score	0.05	0.06	0.43	0.000	0.18	0.18	223.84	0.000^a^
2	Sleep total score	0.05	0	0.39	0.000	0.2	0.02	127.42	0.000^b^
	Epworth sleepiness total score	−0.02	0.01	−0.15	0.000				
3	Sleep total score	0.04	0	0.35	0.000	0.26	0.06	115.91	0.000^c^
	Epworth daytime sleepiness total score	−0.02	0	−0.13	0.000				
	Perceived work safety	0.15	0.02	0.24	0.000				

*Pairwise deletion was performed to treat missing values. ^*a*^df_1_ = 1, df_2_ = 1,017. ^*b*^df_1_ = 2, df_2_ = 1,016. ^*c*^df_1_ = 3, df_2_ = 1,015.*

**FIGURE 3:**
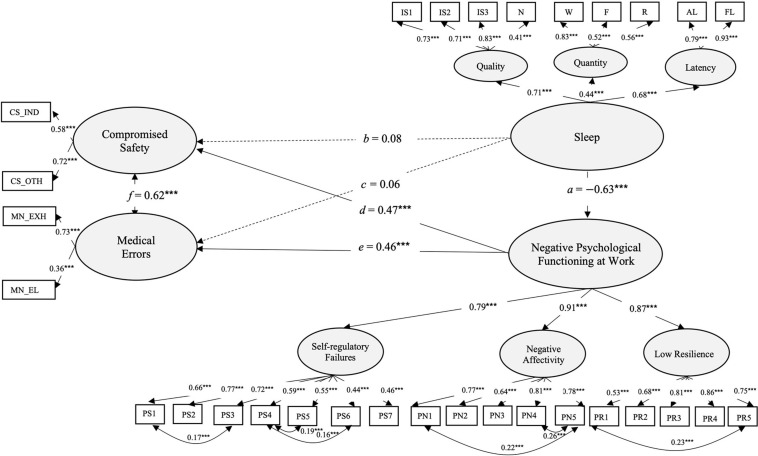
Structural equation model showing the influence of sleep on psychological functioning at work, compromised safety and medical errors. Coefficients represent standardized estimates (*n* = 1,189). Statistical significance levels *p < 0.05, **p < 0.01, ***p < 0.001.

First, we investigated the predictor power of sleep and its effects on psychological functioning at work. To improve the model fit we allowed five covariances and one covariance between latent dimensions. Robust Maximum Likelihood statistics χ^2^ = 1,142.06, *df* = 386, *p* = 0.000, χ^2^/*df* = 2.95, *CFI* = 0.93, *TLI* = 0.92, *RMSEA* = 0.04, *p* = 1, 90% *CI* (0.04, 0.05), *SRMR* = 0.06, showed adequate fit to the hypothesized structure. Structural equation modeling showed that latent dimension of sleep significantly predicted a decrease in negative psychological functioning at work [*a* = −0.63, *p* < 0.001, 95% *CI* (−0.95, −0.66), *B* = −0.8, *SE* = 0.08]. However, contrary to the expectations, there were no significant direct effects of sleep on the incidences of compromised safety [*b* = 0.08, *p* > 0.05, 95% *CI* (−0.08, 0.25), *B* = 0.09, *SE* = 0.09] and medical errors [*c* = 0.06, *p* > *0.05*, 95% *CI* (−0.1, 0.21), *B* = 0.06, *SE* = 0.09]. Negative psychological functioning at work, on the other hand, increased the incidences of medical errors [*d* = 0.46, *p* < 0.001, *B* = 0.4, *SE* = 0.07, 95% *CI* (0.32, 0.6)] and compromised safety [*e* = 0.47, *p* < 0.001, *B* = 0.39, *SE* = 0.07, 95% *CI* (0.27, 0.53)]. Sleep had indirectly, by decreasing negative psychological functioning at work, decreased incidences of medical errors [*ae* = −0.32, *p* < 0.001, *SE* = 0.06, 95% *CI* (−0.39, −0.19)] and compromised safety (*ad* = −0.33, *p* < *0.001*, *B* = −0.32, *SE* = 0.06, 95% *CI* (−0.39, −0.2)]. Significant covariances were found between medical errors and compromised safety [*f* = 0.62, *p* < 0.001, *B* = 0.62, *SE* = 0.08, 95% *CI* (0.47, 0.78)]. This shows, partial support for the hypothesized model, with better sleep directly decreasing negative psychological functioning at work, and by doing so indirectly decreasing the incidences of compromised safety and medical errors (Figure 3).

**FIGURE 4:**
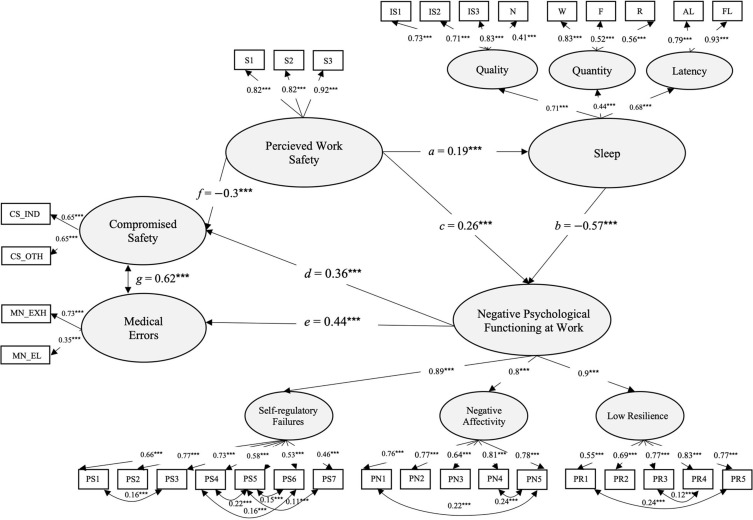
Structural equation model, showing the influence of sleep and perceived work safety on negative psychological functioning at work, compromised safety, and medical errors during the first month of the COVID-19 epidemic. Coefficients represent standardized estimates (*n* = 1,189). Statistical significance levels *p < 0.05, **p < 0.01, ***p < 0.001.

We tested the second model when perceived work safety was added into the model. To improve the model fit, nine covariances between indicators and one on latent dimensions of medical errors and compromised safety. The model was within the recommended standards with Robust Maximum Likelihood statistics χ^2^ = 1,336.27, *df* = 484, *p* = 0.000, χ^2^/*df* 2.77, *CFI* = 0.94, *TLI* = 0.93, *RMSEA* = 0.04, *p* = 1, 90% *CI* (0.04, 0.05), *SRMR* = 0.06, showing adequate fit to the data. Perceived work safety at the time of the Covid-19 epidemic has shown significant improvement in sleep [*a* = 0.19, *p* < *0.001*, 95% *CI* (0.1, 0.28), *B* = 0.19, *SE* = 0.05], and reduction in negative psychological functioning at work [*c* = −0.33, *p* < 0.001, 95% *CI* (−0.33, −0.19), *B* = −0.35, *SE* = 0.05] and incidences of compromised safety [*f* = −0.26, *p* < 0.001, 95% *CI* (−0.33, −0.19), *B* = −0.25, *SE* = 0.05]. Sleep predicted significantly less negative psychological functioning at work [*b* = −0.57, *p* < 0.001, 95% *CI* (−0.64, −0.5), *B* = −0.75, *SE* = 0.07], while negative psychological functioning at work caused a significant increase in medical errors [*e* = 0.31, *p* < 0.001, 95% *CI* (0.27, 0.47), *B* = 0.37, *SE* = 0.05] and compromised safety [*d* = 0.44, *p* < 0.001, 95% *CI* (0.35, 0.54), *B* = 0.31, *SE* = 0.05]. Significant co-variances were found between compromised safety and medical errors [*g* = 0.62, *p* < 0.001, 95% *CI* (0.46, 0.78), *B* = 0.62, *SE* = 0.08]. In the same way, as in the previous model, sleep had indirectly, by decreasing negative psychological functioning at work, increased the likelihood of medical errors [*bd* = −0.26, *p* < 0.001, 95% *CI* (−0.26, −0.14), *B* = −0.28, *SE* = 0.08] and compromised safety [*be* = −0.25, *p* < 0.001, 95% *CI* (−0.32, −0.19), *B* = 0.11, *SE* = 0.02]. Different to the expectations, perceived work safety has shown a small, however significant, indirect effect by decreasing negative psychological functioning on the incidences of medical errors [*cd* = *−0.*12, *p* < 0.001, 95% *CI* (−0.16, −0.06), *B* = −0.1, *SE* = 0.03]. The model supports the hypothesized model, showing perceived work safety as having important direct influence on improving sleep, reducing negative psychological functioning at work, compromised safety, and medical errors (Figure 4).

Binary logistic regression analysis was performed to investigate how sleep, psychological functioning at work, sleepiness, and perceived work safety differed between physicians working at COVID-19 entry point and others. Hosmer and Lemeshow test showed adequate fit to the data χ^2^ (8) = 5.99, *p* = 0.645, explaining 9.2% of total variance (Nagelkerke *R*^2^ = 0.92). Based on Wald statistics, physicians that worked at COVID-19 entry point were 1.26 times more likely to wake up during the night (*p* < 0.05), 1.25 times more likely to experience nightmares (*p* < 0.05), and 0.77 more likely to sleep less than 5 h per night (*p* < 0.01). Physicians working at COVID-19 entry points had significantly lower levels of psychological functioning at work (*p* < 0.001) (Table 5).

**TABLE 5:** Descriptive statistics and binary logistic regression for sleep, psychological functioning at work, sleepiness, and perceived work safety for groups of physicians working at COVID-19 entry point and other physicians (*n* = 1,019).

	COVID-19 entry point physicians *M* (*SD*)	Other physicians *M* (*SD*)	*B*	*SE*	*Wald*	*p*	*Exp (B)*
**Sleep duration (*n* = 1,031)**							
Sleep workdays^a^	0.81 (0.72)	1 (0.78)	−0.13	0.12	1.18	0.277	0.88
Sleep non-workdays^a^	1.87 (0.97)	1.97 (1.04)	0.06	0.08	0.53	0.468	1.06
Average sleep latency^b^	2.38 (0.97)	2.49 (0.85)	0.01	0.12	0.01	0.915	1.01
*f*^c^ (*n* = 1,031)							
…Needed more than 30 min to fall asleep.	1.62 (1.15)	1.8 (1.08)	−0.08	0.1	0.61	0.434	0.92
…Woke up in the middle of the night.	1.09 (1.02)	1.07 (1)	0.23	0.09	5.61	0.018	1.26
…Woke up too early.	1.19 (1.09)	1.38 (1.09)	−0.13	0.1	2.14	0.976	0.88
…Had difficulties falling back asleep after night awakening.	1.62 (1.14)	1.73 (1.08)	0	0.09	0	0.010	1
…Experienced nightmares.	2.12 (0.92)	2.09 (1.07)	0.23	0.09	6.62	0.006	1.25
…Slept less than 5 h.	1.94 (0.92)	2.2 (0.87)	−0.25	0.09	7.41	0.243	0.78
ΣEpworth daytime sleepiness (*n* = 1,019)	7.65 (4.48)	6.84 (4.11)	0.02	0.02	1.36	0.000	1.02
Psychological functioning at work (*n* = 1,189)	3.32 (0.7)	3.53 (0.67)	0.13	0.13	25.7	0.000	0.52
Perceived work safety (*n* = 1,118)	2.91 (1.15)	2.81 (1.09)	0.07	0.07	12.24	0.11	1.28

*Pairwise deletion was performed to treat the missing values. ^*a*^“How long (in hours) have you slept on average per night during the past month? (0—< 6 h, 1—6–7 h, 2—7–8 h, 3—8–9 h, 4—9–10 h, 5— >10 h).” ^*b*^“How much time have you needed on average in the past month to fall asleep? (0—less than 30 min, 1.5—from 30 to 60 min, 3—more than 60 min).” ^*c*^“In the past month, how often has it occurred to you on average…” (3—never, 2—less than once a week, 1—once or twice a week, 0—three or more times a week).*

## Discussion

To our knowledge, no prior studies have investigated how physicians’ sleep and perceived work safety during the first month of the COVID-19 epidemic could have impacted physician psychological functioning at work and the role they had in ensuring patient and physician safety. Physicians working at a COVID-19 entry points were more likely to wake up during the night, have nightmares, and sleep less than 5 h per night. This supports previous findings on medical staff from Wuhan, China, which showed that medical staff working in isolation unit had 1.71 times higher probability of reporting insomnia symptoms (Zhang et al., 2020). Similarly, our findings showing higher incidences of nightmares among healthcare workers working at COVID-19 entry points support previous research that suggests nightmares present one of the symptoms of post-traumatic stress disorder (Campbell and Germain, 2016; Rangachari and Woods, 2020), with healthcare workers working directly with COVID-19 patients reporting significantly more PTSD symptoms in comparison to other healthcare workers (Johnson et al., 2020).

Our results show that the majority of physicians slept less than what is recommended by the American Academy for Sleep Medicine and Sleep Research Society (Watson et al., 2015). Physicians, 28.9%, are under the influence of sleep deprivation on workdays, which is concerning, as previous research suggests that sleep restriction of 6 h per night contributes to cognitive performance deficits equivalent to two nights of total sleep deprivation (Van Dongen et al., 2003). Sleep and perceived work safety, both had a preventative role in ensuring that physicians maintain good levels of psychological functioning at work even during the crisis. Contrary to the expectations, no direct effect was found of sleep on compromised safety and medical errors. Nevertheless, sleep, by decreasing negative psychological functioning at work, decreases incidences of committing adverse and potentially fatal incidents, such as compromised safety and medical errors. Our findings are therefore, only partial in line with previous research linking sleep deprivation to increase in medical errors and compromised safety (Barger et al., 2006; Lockley et al., 2007; Smith and Plunkett, 2019). However, they provide support for theoretical propositions placed forward by Barnes (2012) on sleep involvement in the processes of self-regulation.

Physicians that slept well in the first month of the COVID-19 epidemic experienced less self-regulatory failures at work, had lower negative affectivity, and were able to remain resilient while working. This provides support for previous findings linking sleep to better cognitive and emotional self-regulation (Hagger et al., 2010; Barnes, 2012; Rosales-Lagarde et al., 2012; Krizan and Hisler, 2016; Palmer and Alfano, 2017), decrease in negative affectivity (Zohar et al., 2005; Deliens et al., 2014), and better resilience (Pedersen et al., 2015). Furthermore, our research shows the importance sleep plays in preventing cognitive failures that have shown, similar to our findings, negative impact on safety (Brossoit), as well as in emotional regulation, which works in prevention of self-injury (You et al., 2018) and can provide additional support to models such as Croskerry et al. (2010) that link emotional state of physicians as important in ensuring better judgment, decision making, and patient safety. By testing the hypothesized model, our findings showed that when perceived work safety was added into the second model, the model showed significant improvement, with perceived work safety being linked to better sleep, lower level of negative psychological functioning at work, and higher incidences of compromised safety reported by physicians. These findings support the previous research that linked worries of personal safety and transmitting the disease to family members to reduction in sleep health during the COVID-19 pandemic (Singh et al., 2020). No significant differences were found in physicians’ evaluation of perceived work safety between a group of physicians working at COVID-19 entry point and others.

Our study included a large sample of physicians and carries some important implications in terms of work settings and crisis management. Even though the Sleep and Psychological Functioning at Work Scale requires modifications, further validation and small sensitivity improvements, its psychometric properties, and established construct validity imply good potential for future research and monitoring purposes. By using retrospective self-reports, we were able to reach a large sample of physicians across Slovenia, which would have been otherwise very difficult to obtain due to quarantine restrictions imposed by the government such, as restriction of movement between municipalities and social distancing (Uradni List Rs št 38, 2020). It provided us with an insight into physicians’ subjective perception of sleep, which can still provide a valuable information about sleep (Ibanez et al., 2018). In the interpretation of our findings, there are some limitations to consider. Previous studies show that retrospective self-reports are prone to distortion by memory recall and motives to provide biased responses (Stone et al., 2009), since respondents tend to overestimate sleep duration (Lauderdale et al., 2008), Findings by Van Dongen et al. (2003) suggest that participants are largely unaware of the increasing cognitive deficits in chronic sleep condition (<6 h sleep), which can lead to underreporting in work-related measures and could explain why no direct relationship was found between sleep, medical errors, and compromised safety. To further validate our findings, we suggest that convergent validity is established by comparing our measure and findings with objective measures such as actigraphy (Sadeh, 2011) or results on psychomotor-vigilance task (Wilkinson and Houghton, 1982) that are frequently used in order to objectively measure sleep and its effects (Loh et al., 2004). Our study measured potential cumulative effects based on theoretical propositions and research placed forward by organizational researchers that suggest both sleep quantity and quality play an important role in ensuring self-regulation, as well as optimal states, behaviors, and attitudes at work (Barnes, 2012; Crain et al., 2018; Pilcher and Morris, 2020). It does not, however, differentiate between the effects of sleep on workdays vs. non-workdays, changes in sleep duration, and specific items on sleep quality, such as sleep fragmentation and nightmares in investigating its effects on psychological functioning at work. Our study has not included a sufficient sample of long sleepers in order to investigate the effects of long sleep on psychological functioning at work. Research, for example, shows that sleeping longer than 9 h per night may be appropriate for young adults or individuals recovering from sleep debt (Watson et al., 2015). It can, however, reduce cognitive functioning (Kronholm et al., 2009) and is associated with depression (Patel et al., 2006), which is why we propose future studies on larger sample sizes, recruiting longer sleepers to differentiate for potential effects of long sleep on physicians’ psychological functioning at work.

Based upon our findings, training could be designed that would help physicians, identify and change potential outcomes of cognitive failures, regulate emotions, and remain resilient in difficult situations. Further research is needed, to see how crisis management during the first month of COVID-19 epidemic, could have impacted physicians’ sleep and psychological functioning at work differently, as it would have had in normal circumstances. In the future, special care should be taken to see how medical guidelines can be updated to better protect safety and sleep of physicians.

## Conclusion

Working at Covid-19 entry points increased the likelihood of sleep awakening during the night, nightmares, occurrences of sleep lower than 5 h, and lower psychological functioning at work. However, this can be problematic, as sleep and safety both play an important role in reducing negative psychological functioning at work and, by doing so, decreasing the likelihood that physicians will enact negative and potentially fatal incidents during the pandemic, such as compromised safety and medical errors. Further studies should be taken to see how medical guidelines can be adapted, to ensure physicians receive enough sleep and that their safety is protected.

## Data Availability Statement

All datasets generated for this study are included in the article/Supplementary Material, further inquiries can be directed to the corresponding author/s.

## Ethics Statement

The study was conducted in accordance with the 1964 Declaration of Helsinki and its later amendments. The study was approved by the Executive committee of the Medical Chamber of Slovenia.

## Author Contributions

All authors developed the study concept, contributed to the study design, data collection, and data analysis, interpreted the data, drafted the manuscript, and approved the final version for submission.

## Conflict of Interest

The authors declare that the research was conducted in the absence of any commercial or financial relationships that could be construed as a potential conflict of interest.

## References

[B1] Akerstedt T. Anund A. Axelsson J. Kecklund G. (2014). Subjective sleepiness is a sensitive indicator of insufficient sleep and impaired waking function. *J. Sleep Res.* 23 240–252. 10.1111/jsr.12158 24750198

[B2] Alhola P. Polo-Kantola P. (2007). Sleep deprivation: impact on cognitive performance. *Neuropsychiatr. Dis. Treat.* 3 553–567. 19300585 PMC2656292

[B3] Arrogante O. Aparicio-Zaldivar E. (2017). Burnout and health among critical care professionals: the mediational role of resilience. *Intensive Crit. Care Nurs.* 42 110–115. 10.1016/j.iccn.2017.04.010 28545878

[B4] Artuch-Garde R. Gonzalez-Torres M. C. Fuente J. Vera M. M. Fernandez-Cabezas Lopez-Garcia M. (2017). Relationship between resilience and self-regulation: a study of Spanish youth at risk of social exclusion. *Front. Psychol.* 8:612. 10.3389/fpsyg.2017.00612 28473792 PMC5397523

[B5] Barger L. K. Ayas N. T. Cade B. E. Cronin J. W. Rosner B. Speizer F. E. (2006). Impact of extended shifts on medical errors, adverse events and attentional failures. *PLoS Med.* 3:e487. 10.1371/journal.pmed.0030487 17194188 PMC1705824

[B6] Barnes C. M. (2012). Working in our sleep: sleep and self-regulation in organizations. *Organ. Psychol. Rev.* 2 234–257. 10.1177/2041386612450181

[B7] Baumesiter R. F. Vohs K. D. Tice D. M. (2011). The strength model of self-control. *Curr. Direct. Psychol. Sci.* 16 351–355. 10.1111/j.1467-8721.2007.00534.x

[B8] Brossoit R. M. Crain T. L. Leslie J. J. Hammer L. B. Truxillo D. M. Bodner T. E. (2019). The effects of sleep on workplace cognitive failure and safety. *J. Occup. Health Psychol.* 24 411–422. 10.1037/ocp0000139 30489101 PMC9036851

[B9] Campbell R. L. Germain A. (2016). Nightmares and posttraumatic stress disorder (PTSD). *Curr. Sleep Med. Rep.* 2 74–80. 10.1007/s40675-016-0037-0

[B10] Čebašek-Travnik Z. Lukaè M. Kos I. (2020). *Zbornica Predlaga, Da Vstopne Toèke Organizira NIJZ in v Njih Izvaja Preglede Ter Usmerja Paciente.* Available online at: https://www.zdravniskazbornica.si/informacije-publikacije-in-analize/obvestila/2020/02/26/zbornica-predlaga-da-vstopne-toèke-organizira-nijz-in-v-njih-izvaja-preglede-ter-usmerja-paciente (accessed November, 2020).

[B11] Crain T. L. Brossoit R. M. Fisher G. G. (2018). Work, nonwork and sleep (WNS): a review and conceptual framework. *J. Bus. Psychol.* 33 675–697. 10.1007/s10869-017-9521-x

[B12] Croskerry P. Abbass A. Wu A. W. (2010). Emotional influences in patient safety. *J. Patient Saf.* 6 199–205. 10.1097/pts.0b013e3181f6c01a 21500605

[B13] Deliens G. Gilson M. Peigneux P. (2014). Sleep and the processing of emotions. *Exp. Brain Res.* 232 1403–1414. 10.1037/ocp0000139 24449011

[B14] Eurostat (2019). *Healthcare Personnel Statistics – Physicians.* Available online at: https://ec.europa.eu/eurostat/statistics-explained/pdfscache/37382.pdf (accessed April 29, 2020).

[B15] FIDES (n.d.). *Novinarska Konferenca o Pomanjkanju Zdravnikov in Njihovi Izgorelosti [Press release].* Available online at: https://sindikatfides.si/obvestila/novinarska-konferenca-o-pomanjkanju-zdravnikov-njihovi-izgorelosti (accessed September 20, 2020).

[B16] Gabriel A. S. Diefendorff J. M. Erickson R. J. (2011). The relations of daily task accomplishment satisfaction with changes in negative affect: a multilevel study in nurses. *J. Appl. Psychol.* 95 1095–1104. 10.1037/a0023937 21639600

[B17] Germain A. Dretsch M. (2016). Sleep and resilience – A call for prevention and intervention. *Sleep* 39 1111–1120. 10.5665/sleep.5732 27091522 PMC4835317

[B18] Hagger M. S. Wood C. Stiff C. Chatzisarantis N. L. D. (2010). Ego depletion and the strength model of self-control: a meta-analysis. *Psychol. Bull.* 136 495–525. 10.1037/a0019486 20565167

[B19] Harvey A. Stinson K. Whitaker K. L. Moskovitz D. Virk H. (2008). The subjective meaning of sleep quality: a comparison of individuals with and without insomnia. *SLEEP* 31 383–393. 10.1093/sleep/31.3.383 18363315 PMC2276747

[B20] Huang Y. Zhao N. (2020). Generalized anxiety disorder, depressive symptoms and sleep quality during COVID-19 outbreak in China: a web-based cross-sectional survey. *Psychiatry Res.* 288:112954. 10.1016/j.psychres.2020.112954 PMC7152913 32325383

[B21] Ibanez V. Silva J. Cauli O. (2018). A survey on sleep assessment methods. *Peer J.* 6 1–26. 10.7717/peerj.4849 29844990 PMC5971842

[B22] Jensen P. M. Trollope-Kumar K. Waters H. Everson J. (2008). Building physician resilience. *Can. Fam. Phys. Med. Fam. Canadien* 54 722–729. PMC2377221 18474706

[B23] Johns M. W. (1992). Reliability and factor analysis of the epworth sleepiness scale. *Sleep* 15 376–381. 10.1093/sleep/15.4.376 1519015

[B24] Johnson S. U. Ebrahimi O. V. Hoffart A. (2020). PTSD symptoms among health workers and public service providers during the COVID-19 outbreak. *PLoS One* 15:e0241032. 10.1371/journal.pone.0241032 33085716 PMC7577493

[B25] Karoly P. (1993). Mechanisms of self-regulation: a systems view. *Annu. Rev. Psychol.* 44 23–52. 10.1146/annurev.psych.44.1.23

[B26] Klim J. (n.d.). *IZJAVA ZA JAVNOST - Zdravniki Primarnega Nivoja Opozarjajo Na Nujnost Doloèitve Jasnega Algoritma za Obravnavo Potencialno Obolelih Pacientov s Covid-19 [Press release].* Available online at: https://sindikatfides.si/obvestila/izjava-za-javnost-zdravniki-primarneganivoja-opozarjajo-na-nujnost-dolo%C4%8Ditve-jasnega (accessed September 21, 2020).

[B27] Krizan Z. Hisler G. (2016). “The essential role of sleep in self-regulation,” in *Handbook of Self-Regulation*, 3rd Edn, eds Vohs K. D. Baumeister R. F. (New York: Guilford Press).

[B28] Kronholm E. Sallinen M. Suutama T. Sulkava R. Era P. Partonen T. (2009). Self-reported sleep duration and cognitive functioning in the general population. *J. Sleep Res.* 18 436–446. 10.1111/j.1365-2869.2009.00765.x 19732318

[B29] Lauderdale D. S. Knutson K. L. Yan L. L. Liu K. Rathouz P. J. (2008). Sleep duration: how well do self-reports reflect objective measures? The CARDIA Sleep Study. *Epidemiology* 19 838–845. 10.1097/EDE.0b013e318187a7b0 18854708 PMC2785092

[B30] Litwiller B. Snyder A. L. Taylor W. D. Steele L. M. (2017). The relationship between sleep and work: a meta-analysis. *J. Appl. Psychol.* 102 682–699. 10.1037/apl0000169 27893255

[B31] Lockley S. W. Barger L. K. Ayas N. T. Rothschild J. Czeisler C. A. Landrigan C. P. (2007). Effects of health care provider work hours and sleep deprivation on safety performance. *Joint Commission J. Qual. Patient Saf.* 33 7–18. 10.1016/s1553-7250(07)33109-7 18173162

[B32] Loh S. Lamond N. Jillian J. Roach G. Drew D. (2004). The validity of psychomotor vigilance tasks of less than 10-minute duration. *Behav. Res. Methods Instr. Comput.* 36 339–346. 10.3758/BF03195580 15354700

[B33] Marsh H. W. Hau K.-T. Gray D. (2005). “Goodness of fit in structural equation models,” in *Contemporary Psychometrics*, eds Maydeu-Olivares A. McArdle J. J. (Mahwah, NJ: Lawrence Erlbaum Associates), 276–335.

[B34] McCann C. M. Beddoe E. McCormik K. Huggard P. Kedge S. Adamson C. (2013). Resilience in the health professionals: A review of recent literature. *Int. J. Wellbeing* 3 60–81. 10.5502/ijw.v3i1.4 32285354

[B35] Medic G. Wille M. Hemels M. E. H. (2017). Short- and long-term health consequences of sleep disruption. *Nat. Sci. Sleep* 9 151–161. 10.2147/NSS.S134864 28579842 PMC5449130

[B36] Ministrstvo za zdravje-Republika Slovenija (2020). *Obravnava Bolnika z Akutno Okužbo Dihal v Vstopni Ambulanti za Covid-19.* Available online at: https://www.gov.si/teme/koronavirus-sars-cov-2/za-izvajalce-zdravstvene-dejavnosti/ (accessed November 1, 2020).

[B37] Montero-Marin J. Tops M. Manzanera R. Piva Demarzo M. M. Alvarez de Mon M. Garcia-Campayo J. (2015). Mindfulness, resilience, and burnout subtypes in primary care physicians: the possible mediating role of positive and negative affect. *Front. Psychol.* 6:1895. 10.3389/fpsyg.2015.01895 26733900 PMC4681844

[B38] Motoh H. (2020). Slovenia social briefing: Covid-19 epidemics reveals the weak points of Slovenian healthcare system and related systems. *China CEE Inst. Week. Brief.* 27 1–4.

[B39] NIJZ (2020). *Dnevno spremljanje okužb s SARS-CoV-2 (COVID-19).* Available online at: https://www.nijz.si/sl/dnevno-spremljanje-okuzb-s-sars-cov-2-covid-19 (accessed July 31, 2020).

[B40] Nuzzo J. B. Meyer D. Snyder M. Ravi S. J. Lapascu A. Souleles J. (2019). What makes health systems resilient against infectious disease outbreaks and natural hazards? Results from a scoping review. *BMC Public Health* 19:1310. 10.1186/s12889-019-7707-z 31623594 PMC6798426

[B41] Palmer C. Alfano C. (2017). Sleep and emotion regulation: an organizing integrative review. *Sleep Med. Rev.* 31 6–16. 10.1016/j.smrv.2015.12.006 26899742

[B42] Pappa S. Ntella V. Giannakas T. Giannakoulis V. G. Papoutsi E. Katsaounou P. (2020). Prevalence of depression, anxiety, and insomnia among healthcare workers during the COVID-19 pandemic: a systematic review and meta-analysis. *Brain Behav. Immun.* 88 901–907. 10.1016/j.bbi.2020.05.026 32437915 PMC7206431

[B43] Patel S. R. Malhotra A. Gao Z. Hu F. B. Neuman M. I. Fawzi W. W. (2011). A prospective study of sleep duration and pneumonia risk in women. *Sleep* 35 97–101. 10.5665/sleep.1594 22215923 PMC3242694

[B44] Patel S. R. Malhotra A. Gottlieb D. J. White D. P. Hu F. B. (2006). Correlates of long sleep duration. *Sleep* 29 881–889. 10.1093/sleep/29.7.881 16895254 PMC3500381

[B45] Pedersen E. R. Troxel W. M. Shih R. A. Pinder E. Lee D. Geyer L. (2015). Increasing resilience through promotion of healthy sleep among service members. *Mil. Med.* 180 4–6. 10.7205/MILMED-D-14-00264 25562849 PMC4356633

[B46] Pilcher J. J. Morris D. M. (2020). Sleep and organizational behavior: implications for workplace productivity and safety. *Front. Psychol.* 11:45. 10.3389/fpsyg.2020.00045 PMC7005570 32082218

[B47] Prather A. A. Leung C. W. (2016). Association of insufficient sleep with respiratory infection among adults in the United States. *JAMA Intern. Med.* 176 850–852. 10.1001/jamainternmed.2016.0787 27064773 PMC4899278

[B48] Rangachari P. Woods L. J. (2020). Preserving organizational resilience, patient safety, and staff retention during COVID-19 requires a holistic consideration of the psychological safety of healthcare workers. *Int. J. Environ. Res. Public Health* 17:4267. 10.3390/ijerph17124267 32549273 PMC7345925

[B49] Republika Slovenija Državni Zbor (2019). *ODBOR ZA ZDRAVSTVO: 8. Nujna Seja (12. April 2019).* Available online at: http://www.dz-rs.si/wps/portal/Home/deloDZ/seje/evidenca?mandat=VIII&type=pmagdt&uid=62C897D311A1670EC12583E6002BD7A3 (accessed November 1, 2020).

[B50] Rosales-Lagarde A. Armony J. L. del Rio-Portilla Y. Conde R. Corsi-Cabrera M. (2012). Enhanced emotional reactivity after selective REM sleep deprivation in humans: an FMRI study. *Front. Behav. Neurosci.* 6:25. 10.3389/fnbeh.2012.00025 22719723 PMC3376727

[B51] Sadeh A. (2011). The role and validity of actigraphy in sleep medicine: an update. *Sleep Med. Rev.* 15 259–267. 10.1016/j.smrv.2010.10.001 21237680

[B52] Schweizer K. (2010). Some guidelines concerning the modelling of traits and abilities in test construction. *Eur. J. Psychol. Assess.* 26 1–2. 10.1027/1015-5759/a000001

[B53] Scott B. A. Judge T. A. (2006). Insomnia, emotions, and job satisfaction: a multilevel study. *J. Manag.* 32 622–645. 10.1177/0149206306289762

[B54] Sher L. (2020). Sleep, resilience and suicide. *Sleep Med.* 66 284–285. 10.1016/j.sleep.2019.08.015 31859035

[B55] Singh M. Sharda S. Gautman M. Hawa R. (2020). Optimal sleep health among frontline healthcare workers during the COVID-19 pandemic. *Can. J. Anasth.* 10.1007/s12630-020-01716-2 PMC7232925 32425331

[B56] Smith A. F. Plunkett E. F. (2019). People, systems and safety: resilience and excellence in healthcare practice. *Anaesthesia* 74 508–517. 10.1111/anae.14519 30585298 PMC6766951

[B57] Spira A. P. Beaudreau S. A. Stone K. L. Keziran E. J. Lui L.-Y. Redline S. (2011). Reliability and validity of the pittsburgh sleep quality index and the epworth sleepiness scale in older men. *J. Garentol. Med. Sci.* 67A 433–439. 10.1093/gerona/glr172 21934125 PMC3309871

[B58] Stone A. Turkkan J. S. Bachrach C. A. Jobe J. B. Kurtzman H. S. Cain V. S. (2009). *The Science of Self-Report: Implications for Research and Practice.* Available online at: https://books.google.si/books?id=k9p4AgAAQBAJ&printsec=frontcover&hl=sl#v=onepage&q&f=false (accessed December 7, 2020).

[B59] Sujan M. A. Furniss D. Andersen J. Braithwaite J. Hollnagel E. (2019). Resilient Health Care as the basis for teaching patient safety – A safety-II critique of the World Health Organisation patient safety curriculum. *Saf. Sci.* 118 15–21. 10.1016/j.ssci.2019.04.046

[B60] Uradni List Rs št 38 (2020). *668. Odlok o Zaèasni Splošni Prepovedi Gibanja in Zbiranja Ljudi na Javnih Mestih in Površinah v Republiki Sloveniji ter Prepovedi Gibanja Izven Obèin, 1943.* Available online at: https://www.uradni-list.si/1/objava.jsp?sop=2020-01-0688 (accessed February 1, 2020).

[B61] Van Dongen H. P. Maislin G. Mullington J. M. Dinges D. F. (2003). The cumulative cost of additional wakefulness: dose-response effects on neurobehavioral functions and sleep physiology from chronic sleep restriction and total sleep deprivation. *Sleep* 26 117–126. 10.1093/sleep/26.2.117 12683469

[B62] Walker M. P. (2009). The role of sleep in cognition and emotion. *Ann. N.Y. Acad. Sci.* 1156 168–197. 10.1111/j.1749-6632.2009.04416.x 19338508

[B63] Watson N. F. Badr M. S. Belenky G. Bliwise D. L. Buxton O. M. Buysse D. (2015). Joint consensus statement of the American Academy of Sleep Medicine and Sleep Research Society on the recommended amount of sleep for a healthy adult: methodology and discussion. *Sleep* 38 1161–1183. 10.5665/sleep.4886 26194576 PMC4507722

[B64] Wilkinson R. T. Houghton D. (1982). Field test of arousal: a portable reaction timer with data storage. *Hum. Factors* 24 487–493. 10.1177/001872088202400409 7129455

[B65] World Health Organisation (2020). *Strengthening the Health System Response to Covid-19: Recommendations for the WHO European Region.* Geneva: WHO.

[B66] Xiao H. Zhang Y. Kong D. Li S. Yang N. (2020). The effects of social support on sleep quality of medical staff treating patients with coronavirus disease 2019 (COVID-19) in January and February 2020 in China. *Med. Sci. Monit.* 26:e923549. 10.12659/MSM.923549 32132521 PMC7075079

[B67] You J. Ren Y. Xu Z. Zhilong W. Sian X. Min-Pei L. (2018). Emotional dysregulation and nonsuicidal self-injury: a meta-analytic review. *Neuropsychiatry* 8 10.4172/Neuropsychiatry.1000399

[B68] Zhang C. Yang L. Liu S. Ma S. Wang Y. Cai Z. (2020). Survey of insomnia and related social psychological factors among medical staff involved in the 2019 novel coronavirus disease outbreak. *Front. Psychiatry* 11:306. 10.3389/fpsyt.2020.00306 32346373 PMC7171048

[B69] Zohar D. Tzischinsky O. Epstein R. Lavie P. (2005). The effects of sleep loss on medical residents’ emotional reactions to work events: a cognitive-energy model. *Sleep* 28 47–54. 10.1093/sleep/28.1.47 15700720

