# Supplementary material for: Sleep and Safety Improve Physicians’ Psychological Functioning at Work During Covid-19 Epidemic
Source: Front Psychol. 2021 Feb 15;11:569324. doi: 10.3389/fpsyg.2020.569324 (PMC7917138; doi:10.3389/fpsyg.2020.569324)
Supplement: Supplementary file 1 [file Table_1.docx]

*Questionnaire of Sleep and Psychological Functioning at Work*

# APPENDIX 1 | Sleep Scale–Short version

1. How long (in hours) in the past month did you sleep on average per night (time of sleep can be different than the time spent in bed with the purpose of sleep)?
2. [W] Work days:
   1. Less than 6 h
   2. 6–7 h
   3. 7–8 h
   4. 8–9 h
   5. 9–10h
   6. More than 10 h
3. [F] Non-work days:
   1. Less than 6 h
   2. 6–7 h
   3. 7–8 h
   4. 8–9h
   5. 9–10h
   6. More than 10h
4. [AL] How long did you need on average in the past month to fall asleep after turning the light off?
5. Less than 30 min
   1. From 30 to 60 min

3) More than 60 min

1. Statements below refer to sleep. In the past month, how often has it occurred to you on average that you...

|  |  |  | Never | Less than once a week | Once or twice a week | Three or more times a week |
| --- | --- | --- | --- | --- | --- | --- |
| [FL] | …needed more than 30 minutes to fall asleep | |  |  |  |  |
| [IS1] | ...woke up in the middle of the night. | |  |  |  |  |
| [IS2] | ... woke up too early | |  |  |  |  |
| [IS3] | ... experienced difficulties falling back asleep after night awakening | |  |  |  |  |
| [N] | ... experienced nightmares | |  |  |  |  |
| [R] | ... slept less than 5 h | |  |  |  |  |

*Letters in square brackets represent legends used in the paper.

**Scoring:** Sum of scores is used for this scale on dimensions and total scores. To allow for comparability of different weights of items on sleep quantity and latency dimensions the scores of items R, AL are transformed.

**Total score** (sleep quantity + quality + latency) score range from 0 to 30.

- **Sleep quantity:** (W + F + R): score range from 0 to 15.
  - Sleep duration workdays [W] : (0—*< 6 h*, 1—*6–7 h*, 2—*7–8 h*, 3—*8–9 h*, 4—*9–10 h*, 5—*>10 h*).
  - Sleep duration non-workdays [F]: (0—*< 6 h*, 1—*6–7 h*, 2—*7–8 h*, 3—*8–9 h*, 4—*9–10 h*, 5—*>10 h*).
  - Reduced sleep (<5 h) [R]: (0—*never*, 1.67—*less than once a week*, 3.33—*once or twice a week*, 5—*three or more times a week*).
- **Sleep quality:** (IS1 + IS2 + IS3 + N): score range from 0 to 16.
  - 0—*never*, 1—*less than once a week*, 2—*once or twice a week*, 3—*three or more times a week*.
- **Sleep latency:** (AL + FL): score range from 0 to 6.
  - Average sleep latency [AL]: (0—*less than 30 min*, 1.5—*30–60 min,* 3—*>60 min*).
  - Frequency of longer sleep latency [FL]: (0—*never*, 1—*less than once a week*, 2—*once or twice a week*, 3—*three or more times a week*).

# APPENDIX 2 | Psychological Functioning at Work Scale

Statements below refer to your feelings and functioning at work in the past month. Please, mark how often in the past month did/were you...

|  |  | 1.Never | 2.Rarely | 3.Sometimes | 4.Often | 5.Very often |
| --- | --- | --- | --- | --- | --- | --- |
|  | **Self-regulatory failures** | | | | | |
| [PS1] | ... have memory problems. |  |  |  |  |  |
| [PS2] | ... have difficulties making decisions. |  |  |  |  |  |
| [PS3] | ... have difficulties maintaining attention on work activities you were performing. |  |  |  |  |  |
| [PS4] | ... miss something or not see something important. |  |  |  |  |  |
| [PS5] | ... in interaction with others said something you later regretted. |  |  |  |  |  |
| [PS6] | ... make a risky choice. |  |  |  |  |  |
| [PS7] | ... remain indifferent in a situation that required empathy. |  |  |  |  |  |
|  | **Negative affectivity** | | | | | |
| [PN1] | ... feel sadness. |  |  |  |  |  |
| [PN2] | ... feel anger. |  |  |  |  |  |
| [PN3] | ... feel powerless. |  |  |  |  |  |
| [PN4] | ... feel fear. |  |  |  |  |  |
| [PN5] | ... feel concerned. |  |  |  |  |  |
|  | **Resilience** | | | | | |
| [PR1] | ... organized and effective. |  |  |  |  |  |
| [PR2] | ... adapt to changes happening around you with ease. |  |  |  |  |  |
| [PR3] | ... think positively. |  |  |  |  |  |
| [PR4] | ... feel strong and capable when faced with difficulties |  |  |  |  |  |
| [PR5] | ... have enough energy to handle work tasks. |  |  |  |  |  |

*Letters in square brackets represent legends used in the paper.

**Scoring:** Scoring of the items allows both assessing positive or negative end of the continuum.

**Negative psychological functioning at work:**

**Dimensions:**

- Self-regulatory failures: (PS1 + PS2 + PS3 + PS4 + PS5 + PS6 + PS7)/7
- Negative affectivity: (PN1 + PN2 + PN3 + PN4 + PN5)/5
- Low resilience: (6-PR1) + (6-PR2) + (6-PR3) + (6-PR4) + (6-PR5)/5

**Average score:**

- (self-regulatory failures + negative affectivity + low resilience)/3

**Psychological functioning at work:**

**Dimensions:**

- Self-regulation: (6-PS1) + (6-PS2) + (6-PS3) + (6-PS4) + (6-PS5) + (6-PS6) + (6-PS7)/7
- Low negative affectivity: (6-PN1) + (6-PN2) + (6-PN3) + (6-PN4) + (6-PN5)/5
- Resilience: (PR1 + PR2 + PR3 + PR4 + PR5)/5

**Average score:**

- (self-regulation + low negative affectivity + resilience)/3
