# Supplementary material for: Sleep and Safety Improve Physicians’ Psychological Functioning at Work During Covid-19 Epidemic
Source: Front Psychol. 2021 Feb 15;11:569324. doi: 10.3389/fpsyg.2020.569324 (PMC7917138; doi:10.3389/fpsyg.2020.569324)
Supplement: Supplementary file 2 [file Table_2.docx]

***Psychometric Analysis for the Questionnaire Sleep and Psychological Functioning at Work***

Analysis in terms of reliability, as well as correlations on specific dimensions can be found in the article. The code for the psychometric analysis is available on GitHub, some item naming in the code may differ to the dataset so make sure to contact the authors, if you encounter any difficulties: <https://github.com/Ninazupancic/psychfsleep>

Extensive information about development of the scale and in-depth version of psychometric analysis can be found in Slovenian:

Zupancic, N. (2020). Spanje in delo zdravnikov v času epidemije covida-19 (Master Thesis). University of Ljubljana, Ljubljana, Slovenia. DOI: 10.13140/RG.2.2.34274.35521

1. **PSYCHOMETRIC ANALYSIS: SLEEP SCALE**
   1. **Descriptive statistics**

Looking at descriptive statistics, asymmetry and kurtosis coefficients, as well as Kolmogorov-Smirnov and Shapiro-Wilk tests of normality show statistical significance, which all indicate non-normal distribution of the variables. With some variables showing greater abnormal distribution than others (**Table 1**). Nevertheless, the tests can be prone to overly reject the null hypothesis of normal distribution, which is why we’ve used normality plots to investigate the normality of the distribution, which has shown significant deviations from normality on several items. Specifically, looking at Asymmetry and Kurtosis coefficients, we can see that items referring to average sleep latency and wakefulness at work all show the negative asymmetry of the distribution. This can be especially, problematic as we know that asymmetry coefficients tend to be distorted by sample sizes. The tendency for abnormal distribution also is shown based on kurtosis coefficients above one on items referring to longer than 30 minutes sleep latency, difficulties falling back asleep after night awakening, and early waking onset. Items referring to medicine, pauses of breathing and snoring showed high asymmetric and kurtosis distributions.

| **Table 1.**  Mean, Standard deviation, Asymmetry, Kurtosis and normal distribution tests. | | | | | | | | |
| --- | --- | --- | --- | --- | --- | --- | --- | --- |
|  | M | SD | Asymmetry | Kurtosis | Min | Max | Kolmogorov-Smirnov^d^ | Shapiro-Wilk |
| Sleep quantity |  |  |  |  |  |  |  |  |
| Work days | 0.94 | 0.77 | 0.69 | 0.87 | 0 | 5 | 0.27*** | 0.82*** |
| Free days | 1.94 | 1.05 | 0.33 | 0.16 | 0 | 5 | 0.2*** | 0.91*** |
| Sleep latency |  |  |  |  |  |  |  |  |
| Average sleep latency | 2.46 | 0.89 | −1.43 | 0.98 | 0 | 3 | −0.43*** | 0.62*** |
| *f* ^c^ |  |  |  |  |  |  |  |  |
| > 30 min sleep latency | 1.94 | 1.05 | −0.34 | −1.22 | 0 | 3 | −0.2*** | 0.85*** |
| Night awakening | 2.46 | 0.89 | 0.48 | −0.93 | 0 | 3 | −0.22*** | 0.84*** |
| Early waking onset | 1.75 | 1.10 | 0.19 | −1.28 | 0 | 3 | 0.19*** | 0.86*** |
| Difficulties falling back asleep after night awakening | 1.07 | 1.01 | −0.25 | −1.25 | 0 | 3 | 0.19*** | 0.85*** |
| Nightmares | 1.33 | 1.09 | −0.72 | −0.52 | 0 | 3 | 0.26*** | 0.81*** |
| Insufficient sleep | 1.7 | 1.09 | 0.26 | −1.09 | 0 | 3 | 0.19*** | 0.86*** |
| <5 h sleep | 2.13 | .89 | −0.75 | −0.3 | 0 | 3 | 0.25*** | 0.82*** |
| Wakefulness at work | 1,25 | 1.02 | −1.40 | 1.17 | 0 | 3 | 0.37*** | 0.69*** |
| Eliminated items |  |  |  |  |  |  |  |  |
| Subjective sleep quality | 2.23 | 1.05 | −0.1 | −0.72 | 0 | 4 | 0.2*** | 0.91*** |
| Medicine | 2.13 | 0.89 | −3.14 | 9.33 | 0 | 3 | 0.51*** | 0.39*** |
| Pauses of breathing | 2.09 | 1.15 | −4.97 | 26.51 | 0 | 3 | 0.53*** | 0.39*** |
| Snoring | 2.23 | 1.05 | −3.14 | −9.33 | 0 | 3 | 0.51*** | 0.25*** |

**Table 2,** shows correlational analysis between items on Sleep scale. There are small to medium positive correlations present among most variables on sleep scale, indicating internal validity of the measure. The smallest and in some instances insignificant correlations were present for items referring to sleep apnea (pauses of breathing and snoring). Cronbach α change in case of deleted item shows that reliability statistics remains the same or changes in case the items assessing sleep apnea (α = 0.85), pauses of breathing (α = 0.85), medicine taking (α=.84) and days of sleep on free days (α = 0.84). Reliability coefficient decreases in case of eliminated item, which refers to problems falling asleep after nightime awakening (α=.81) and subjective sleep quality (α = 0.81). Inter-item correlations furthermore show good overall discriminant validity with most items above *r_i_*_SCj_ = 0.3. Small discriminant validity of items referring to sleep apnea (*r*_iSCj_ = 0.19), this is in line with previous statistics showing small or insignificant correlations between items of sleep apnea and most items on the scale (*r* = 0.06 - 0.27). This indicated that it might be more suitable to eliminate the items of sleep apnea from our scale development. Subjective sleep quality showed the highest corrected inter-item correlation (*r*_iSC_ = 0.75). Very high discriminant coefficient can mean that the item does not discriminate well from other items measuring the construct, which could be problematic and indicating that the item may be highly correlated with the overall score on the scale.

**Table 2.**

*Spearman-ro correlation coefficients, Cronbach α, if item deleted in brackets and corrected inter-item correlation (r_ISC_).*

|  |  | 1 | 2 | 3 | 4 | 5 | 6 | 7 | 8 | 9 | 10 | 11 | 12 | 13 | 14 | 15 | *r*_iScj_ | |
| --- | --- | --- | --- | --- | --- | --- | --- | --- | --- | --- | --- | --- | --- | --- | --- | --- | --- | --- |
|  | Quantity of sleep |  |  |  |  |  |  |  |  |  |  |  |  |  |  |  |  |  |
| 1 | Sleep workdays | (0.83) |  |  |  |  |  |  |  |  |  |  |  |  |  |  | .42 |  |
| 2 | Sleep non-work days | 0.46*** | (0.84) |  |  |  |  |  |  |  |  |  |  |  |  |  | .32 |  |
| 3 | <5 h sleep | 0.5*** | 0.23*** | (0.83) |  |  |  |  |  |  |  |  |  |  |  |  | .61 |  |
|  | Sleep latency |  |  |  |  |  |  |  |  |  |  |  |  |  |  |  |  |  |
| 4 | Average sleep latency | 0.25*** | 0.16*** | 0.22*** | (0.83) |  |  |  |  |  |  |  |  |  |  |  | .51 |  |
| 5 | >30 min sleep latency | 0.25*** | 0.15*** | 0.24*** | 0.73*** | (0.82) |  |  |  |  |  |  |  |  |  |  | .56 |  |
|  | Sleep quality/Insomnia |  |  |  |  |  |  |  |  |  |  |  |  |  |  |  |  |  |
| 6 | Night-time awaking | 0.18*** | 0.2*** | 0.18*** | 0.31*** | 0.35*** | (0.82) |  |  |  |  |  |  |  |  |  | .57 |  |
| 7 | Early waking onset | 0.22*** | 0.32*** | 0.19*** | 0.28*** | 0.3*** | 0.54*** | (0.82) |  |  |  |  |  |  |  |  | .56 |  |
| 8 | Difficulties falling asleep after night awakening | 0.23*** | 0.28*** | 0.22*** | 0.38*** | 0.46*** | 0.62*** | 0.6*** | (0.81) |  |  |  |  |  |  |  | .67 |  |
|  | Fitful sleep |  |  |  |  |  |  |  |  |  |  |  |  |  |  |  |  |  |
| 9 | Nightmares | 0.1** | 0.04 | 0.15*** | 0.22*** | 0.28*** | 0.33*** | 0.28*** | 0.3*** | (0.83) |  |  |  |  |  |  | .39 |  |
| 10 | Unrested upon waking | 0.36*** | 0.17*** | 0.35*** | 0.33*** | 0.4*** | 0.41*** | 0.36*** | 0.45*** | 0.41*** | (0.82) |  |  |  |  |  | .61 |  |
| 11 | Wakefulness at work | 0.19*** | 0.04 | 0.22*** | 0.16*** | 0.18*** | 0.17*** | 0.19*** | 0.21*** | 0.22*** | 0.35*** | (0.83) |  |  |  |  | .43 |  |
|  | Eliminated items |  |  |  |  |  |  |  |  |  |  |  |  |  |  |  |  |  |
| 12 | Medicine taking | 0.13*** | 0.1** | 0.17*** | 0.12*** | 0.21*** | 0.19*** | 0.2*** | 0.23*** | 0.11*** | 0.18*** | 0.14*** | (0.84) |  |  |  | .3 |  |
| 13 | Subjective sleep quality | 0.43*** | 0.32*** | 0.36*** | 0.48*** | 0.51*** | 0.55*** | 0.5*** | 0.59*** | 0.37*** | 0.61*** | 0.27*** | 0.27*** | (0.81) |  |  | .76 |  |
| 14 | Pauses of breathing during sleep | 0.08* | 0.16*** | 0.14*** | 0.03 | 0.04 | 0.13*** | 0.14*** | 0.14*** | 0.08** | 0.09** | 0.09** | 0.05 | 0.13*** | (0.85) |  | .19 |  |
| 15 | Snoring | 0.1** | 0.06 | 0.15*** | 0.04 | 0.07* | 0.11** | 0.1** | 0.15*** | 0.1** | 0.11*** | 0.12*** | 0.1** | 0.11*** | 0.27*** | (0.84) | .19 |  |

Notes. **p<0.05*, ***p<0.01*, ****p<0.001*, Total Cronbach α=0.84

- 1. **Exploratory factor analysis**

Based on initial analysis showing significant deviations from normality, we’ve used the Unweighted Least Squares method with Keiser Oblimin rotation to investigate the potential factor structure of the scale.


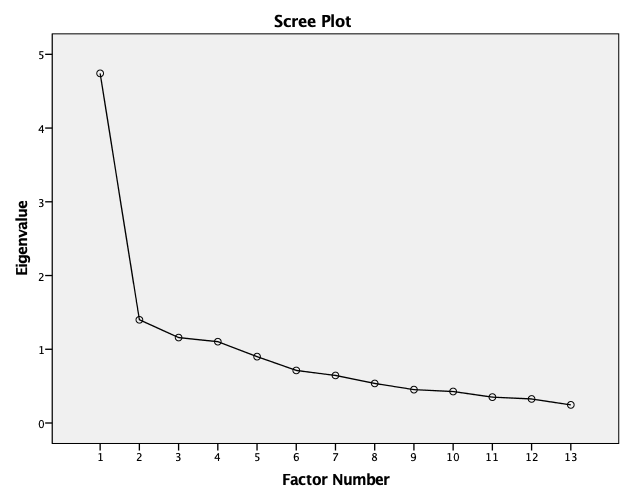


**Figure 1.** Scree plot for Sleep Scale

As it can be seen from Figure 1, there is an indication of potential one higher order factor loading with two to three smaller order factors still above the 1 Eigen value. In fact, as it can be seen from Table 3, the values above 1 indicate potential four factor structure of the scale, explaining 51% of the total variance.

| **Table 3.**  *Eigen values on Sleep Scale* | | | | | | | |
| --- | --- | --- | --- | --- | --- | --- | --- |
| Factor | Initial Eigenvalues | | | Extraction Sums of Squared Loadings | | | Rotation Sums of Squared Loadings^a^ |
|  | Total | % of Variance | Cumulative % | Total | % of Variance | Cumulative % | Total |
| 1 | 4.74 | 36.48 | 36.48 | 4.33 | 33.29 | 33.29 | 3.17 |
| 2 | 1.4 | 10.77 | 47.24 | 0.94 | 7.26 | 40.54 | 1.82 |
| 3 | 1.16 | 8.91 | 56.16 | 0.8 | 6.18 | 46.73 | 3.01 |
| 4 | 1.1 | 8.49 | 64.64 | 0.56 | 4.27 | 51 | 2.45 |
| 5 | .9 | 6.93 | 71.57 |  |  |  |  |
| 6 | .71 | 5.48 | 77.05 |  |  |  |  |
| 7 | .65 | 4.96 | 82.01 |  |  |  |  |
| 8 | .54 | 4.13 | 86.14 |  |  |  |  |
| 9 | .45 | 3.48 | 89.62 |  |  |  |  |
| 10 | .43 | 3.29 | 92.9 |  |  |  |  |
| 11 | .35 | 2.7 | 95.6 |  |  |  |  |
| 12 | .33 | 2.51 | 98.12 |  |  |  |  |
| 13 | .25 | 1.89 | 100 |  |  |  |  |
| Extraction Method: Unweighted Least Squares. | | | | | | | |
| a. When factors are correlated, sums of squared loadings cannot be added to obtain a total variance. | | | | | | | |

Pattern matrix shows, four factor structure as the most optimal factor structure. One of the problematic items, seems to be medicine taking. The first factor of sleep quality referring to insomnia symptoms such as night awakening, early waking onset and difficulties falling asleep after waking. Second factor included items assessing sleep quantity (sleep workdays, sleep non-workdays and frequency of sleeping less then 5hours). Third factor sleep latency refers to the items assessing average sleep latency and prolonged sleep latency. The final factor referred to fitful sleep, such as nightmares, difficulties staying awake at work and feeling unrested upon awakening. The item referring to the frequency of taking the medicine to fall asleep, does not show high factor loading on any of the four factors. This may indicate that the item could be eliminated from the scale. Small factor loading on item of subjective sleep quality referring to both sleep quality factor or fitful sleep factor loading is in line with previous findings showing high corrected inter item correlation. This may indicate that the item might be evaluating more of an overall sleep and be less referring to the specific dimensions of the scale. Third factor refers to sleep latency (average sleep latency and frequency of sleep latency longer than 30 minutes). Fourth factor of fitful sleep is rather interesting. When adding the items assessing the unrested upon waking and ability to maintain wakefulness at work, we hypothesised that the items may refer to sleep quality. Nevertheless, it may seem to be more of a separate factor, which referring to fitful sleep. In fact, research by Paul, Schredl, & Alpers (2015) showed that polysomnographic recordings revealed no difference in overall sleep architecture, sleep cycle duration and REM density and REM duration in each cycle. The authors conclude that nightmares are more of an independent form of disturbed sleep architecture. This may provide support for including the item of nightmares with the sleep dimension of fitful sleep, as its association with impairment suggest that nightmares may affect ones functioning throughout the day.

| **Table 4.**  *Pattern Matrix for Sleep scale Direct Oblimin rotation Unweighted least squares* | | | | |
| --- | --- | --- | --- | --- |
|  | Factor | | | |
|  | Sleep quality  /insomnia | Sleep quantity | Sleep latency | Fitful sleep |
| Sleep workdays | −0.09 | **0.78** | −0.06 | 0.15 |
| Sleep non-workdays | 0.26 | **0.57** | 0.01 | −0.2 |
| Average sleep latency | −0.06 | −0.01 | −**0.93** | −0.07 |
| *f* |  |  |  |  |
| >30min sleep latency | 0.04 | −0.03 | **−0.84** | 0 |
| Night awakening | **0.7** | −0.08 | −0.03 | 0.12 |
| Early waking onset | **0.71** | 0.08 | 0.02 | 0.01 |
| Difficulties falling asleep after night-time awakening | **0.72** | 0.03 | −0.12 | 0.05 |
| Nightmares | 0.2 | −0.14 | −0.04 | **0.43** |
| Unrested upon waking | 0.18 | 0.09 | −0.07 | **0.6** |
| Difficulties staying awake at work | 0.01 | 0.04 | −0.01 | **0.47** |
| <5h sleep | −0.08 | 0.**43** | −0.06 | 0.36 |
| Medicine | 0.12 | 0.05 | −0.13 | 0.12 |
| Subjective sleep quality | 0.38 | 0.19 | −0.21 | 0.34 |
| *Notes.* Extraction Method: Unweighted Least Squares.  Rotation Method: Oblimin with Kaiser Normalization. | | | | |
| a. Rotation converged in 11 iterations. | | | | |

- 1. **Confirmatory factor analysis**

While performing confirmatory factor analysis the items of subjective sleep quality and medicine taking showed significant decrease in model fit and high modification indices with items on other factors, which is why the two items were eliminated from final factor structure. Upon exploratory factor analysis and upon the consultation with a somnologist, we’ve decided to test two forms of a questionnaire. The first form of the questionnaire was a shorter version, including three factor structure without including the potential consequences reduced sleep. This version was used for the purposes of our study “Sleep and Safety Improve Physicians’ Psychological Functioning at Work During Covid-19 Epidemic”. The second form was a longer version including also the consequences of reduced sleep by including the fourth factor of fitful sleep. The confirmatory factor analysis as shown in **Table 5**, has shown the adequate fit for the three-factor structure for the short version of the questionnaire and four-factor structure for the longer version of the questionnaire. No significant differences were found between the hierarchical and non-hierarchical model, which is why we’ve used the hierarchical structure, as the version that fitted better for our analysis and questions asked for the purposes of our study.

**Table 5.**

*Confirmatory factor analysis for Robust Maximum Likelihood for Sleep Scale – short version*

|  | *χ^2^* | *df* | *χ^2^/df* | *p* | *CFI* | *TLI* | *RMSEA (90% CI)* | *p* | *SRMR* |
| --- | --- | --- | --- | --- | --- | --- | --- | --- | --- |
| Sleep scale – short version |  |  |  |  |  |  |  |  |  |
| 1 – factor model | 956.7 | 27 | 35.43 | 0.000 | 0.63 | 0.5 | 0.18 (0.17; 0.19) | 0.000 | 0.12 |
| 2 – factor model | 674.47 | 26 | 25.94 | 0.000 | 0.74 | 0.64 | 0.16 (0.15; 0.17 ) | 0.000 | 0.08 |
| 3 – factor model | 124.58 | 24 | 5.19 | 0.000 | 0.96 | 0.94 | 0.06 (0.05; 0.08) | 0.000 | 0.05 |
| Sleep Scale – longer version |  |  |  |  |  |  |  |  |  |
| 1 – factor model | 956.7 | 27 | 35.43 | 0.000 | 0.63 | 0.5 | 0.18 (0.17; 0.19) | 0.000 | 0.08 |
| 2 – factor model | 918.5 | 53 | 17.33 | 0.000 | 0.78 | 0.73 | 0.13 (0.12; 0.13) | 0.000 | 0.08 |
| 4 – factor model | 192.5 | 38 | 5.07 | 0.000 | 0.95 | 0.93 | 0.06 (0.05; 0.07) | 0.000 | 0.05 |

1. **PSYCHOMETRIC ANALYSIS: PSYCHOLOGICAL FUNCTIONING AT WORK SCALE**
   1. **Descriptive statistics**

From **Table 6**, it can be seen that several items on the scale of Psychological Functioning at Work statistically significantly differ from normality on normal distribution statistics. Nevertheless, normality distribution coefficients tend to overly reject normal distribution in large sample sizes. Looking at assytemtric and kurtosis coefficients, they show small deviations from normality, however the assymetry and kurtosis coefficients tend to be lower in large sample sizes, such as ours, which may still be an indicator of abnormal distribution. Looking at graphs, most items seem to show normal distribution. Nevertheless, still certain items significantly differ from normality (»...been organised and effective«). Although items on Psychological Functioning at Work scale do differ from normal distribution to smaller extent, we propose to use parametric tests to assess the items on this scale.

| **Tabele 6.**  *Descriptive statistics, asymmetry, kurtosis coefficients and coefficients of normal distribution on Psychological Functioning at Work Scale* (*n* = 1189). | | | | | | | | | | |
| --- | --- | --- | --- | --- | --- | --- | --- | --- | --- | --- |
|  | *M* | *SD* | *Mdn* | *Min* | *Max* | Assymmetry | Kurtosis | Kolgomgorov-Smirnov^b^ | Shapiro-Wilk |  |
| PS1 | 2.22 | 0.95 | 2 | 1 | 5 | 0.39 | −0.46 | 0.22*** | 0.88*** |  |
| PS2 | 2.40 | 0.97 | 2 | 1 | 5 | 0.32 | −0.41 | 0.22*** | 0.89*** |  |
| PS3 | 2.39 | 1.01 | 2 | 1 | 5 | 0.38 | −0.48 | 0.22*** | 0.89*** |  |
| PS4 | 2.21 | 0.80 | 2 | 1 | 5 | 0.49 | 0.39 | 0.28*** | 0.85*** |  |
| PS5 | 2.15 | 0.94 | 2 | 1 | 5 | 0.57 | −0.06 | 0.23*** | 0.87*** |  |
| PS6 | 2.24 | 1.02 | 2 | 1 | 5 | 0.60 | −0.20 | 0.23*** | 0.88*** |  |
| PS7 | 2.17 | 1.01 | 2 | 1 | 5 | 0.57 | −0.29 | 0.22*** | 0.87*** |  |
| PN1 | 2.85 | 1.18 | 3 | 1 | 5 | 0.04 | −0.87 | 0.16*** | 0.92*** |  |
| PN2 | 2.80 | 1.16 | 3 | 1 | 5 | 0.08 | −0.85 | 0.16*** | 0.91*** |  |
| PN3 | 2.91 | 1.16 | 3 | 1 | 5 | 0.08 | −0.87 | 0.18*** | 0.92*** |  |
| PN4 | 2.87 | 1.16 | 3 | 1 | 5 | 0.08 | −0.87 | 0.18*** | 0.92*** |  |
| PN5 | 3.44 | 1.08 | 4 | 1 | 5 | −0.24 | −0.78 | 0.21*** | 0.90*** |  |
| PR1 | 3.81 | 0.92 | 4 | 1 | 5 | −0.90 | 0.85 | 0.31*** | 0.84*** |  |
| PR2 | 3.27 | 1.04 | 3 | 1 | 5 | −0.21 | −0.64 | 0.21*** | 0.91*** |  |
| PR3 | 3.64 | 0.96 | 4 | 1 | 5 | −0.43 | −0.32 | 0.25*** | 0.88*** |  |
| PR4 | 3.45 | 0.97 | 4 | 1 | 5 | −0.36 | −0.39 | 0.24*** | 0.89*** |  |
| PR5 | 3.56 | 0.96 | 4 | 1 | 5 | −0.44 | −0.27 | 0.26*** | 0.89*** |  |
| Eliminated items |  |  |  |  |  |  |  |  |  |  |
| PN6 | 3.16 | 1.15 | 3 | 1 | 5 | −0.14 | −0.84 | 0.19*** | 0.91*** |  |
| PS8 | 3.31 | 0.94 | 3 | 1 | 5 | −0.18 | −0.46 | 0.21*** | 0.90*** |  |

- 1. **Internal validity, reliability**

**Table 7**, shows that there were medium to high positive correlations on most items referring to negative psychological functioning at work. In line with propositions, items referring to positive end of the psychological functioning at work, show negative correlations with those referring to negative psychological functioning at work. When performing Cronbach α analysis, the items referring to resilience were reversed in scoring. Corrected inter-item correlation on the other hand, shows high discriminant coefficients on all items above .3. With none of the items reaching the threshold of 0.85.

| **Table 7.**  *Pearson correlations, Cronbach alpha, if item deleted in brackets (* α^−^*) and corrected inter item correlation (r_ISC_) for Psychological Functioning at Work Scale* | | | | | | | | | | | | | | | | | | | |
| --- | --- | --- | --- | --- | --- | --- | --- | --- | --- | --- | --- | --- | --- | --- | --- | --- | --- | --- | --- |
|  | PS1 | PS2 | PS3 | PS4 | PS5 | PS6 | PS7 | PS8 | PN1 | PN2 | PN3 | PN4 | PN5 | PR1 | PR2 | PR3 | PR4 | PR5 | *r_iSc_* |
| PS1 | (0.93) |  |  |  |  |  |  |  |  |  |  |  |  |  |  |  |  |  | −0.42^***^ |
| PS2 | 0.55^***^ | (0.93) |  |  |  |  |  |  |  |  |  |  |  |  |  |  |  |  | −0.44^***^ |
| PS3 | 0.58^***^ | 0.6^***^ | (0.93) |  |  |  |  |  |  |  |  |  |  |  |  |  |  |  | −0.49^***^ |
| PS4 | 0.41^***^ | 0.54^***^ | 0.52^***^ | (0.93) |  |  |  |  |  |  |  |  |  |  |  |  |  |  | −0.46^***^ |
| PS5 | 0.44^***^ | 0.47^***^ | 0.44^***^ | 0.37^***^ | (0.93) |  |  |  |  |  |  |  |  |  |  |  |  |  | −0.33^***^ |
| PS6 | 0.39^***^ | 0.4^***^ | 0.35^***^ | 0.35^***^ | 0.47^***^ | (0.93) |  |  |  |  |  |  |  |  |  |  |  |  | −0.33^**^ |
| PS7 | 0.3^***^ | 0.32^***^ | 0.32^***^ | 0.29^***^ | 0.35^***^ | 0.37^***^ | (0.93) |  |  |  |  |  |  |  |  |  |  |  | −0.3^***^ |
| PS8 | −0.32^***^ | −0.42^***^ | −0.41^***^ | −0.42^***^ | −0.31^***^ | −0.38^***^ | −0.31^***^ | (0.93) |  |  |  |  |  |  |  |  |  |  | 0.54^***^ |
| PN1 | 0.24^***^ | 0.35^***^ | 0.27^***^ | 0.28^***^ | 0.37^***^ | 0.35^***^ | 0.29^***^ | −0.27^***^ | (0.92) |  |  |  |  |  |  |  |  |  | −0.21^***^ |
| PN2 | 0.36^***^ | 0.42^***^ | 0.43^***^ | 0.63^***^ | 0.32^***^ | 0.41^***^ | 0.3^***^ | −0.38^***^ | 0.34^***^ | (0.93) |  |  |  |  |  |  |  |  | −0.45^***^ |
| PN3 | 0.31^***^ | 0.33^***^ | 0.36^***^ | 0.45^***^ | 0.28^***^ | 0.4^***^ | 0.32^***^ | −0.38^***^ | 0.36^***^ | 0.54^***^ | (0.93) |  |  |  |  |  |  |  | −0.35^***^ |
| PN4 | 0.41^***^ | 0.51^***^ | 0.5^***^ | 0.61^***^ | 0.35^***^ | 0.41^***^ | 0.33^***^ | −0.45^***^ | .32^***^ | 0.63^***^ | 0.54^***^ | (0.93) |  |  |  |  |  |  | −0.52^***^ |
| PN5 | 0.42^***^ | 0.43^***^ | 0.45^***^ | 0.5^***^ | 0.35^***^ | 0.36^***^ | 0.36^***^ | −0.37^***^ | 0.32^***^ | 0.52^***^ | 0.45^***^ | 0.56^***^ | (0.92) |  |  |  |  |  | −0.52^***^ |
| PN6 | 0.37^***^ | 0.47^***^ | 0.46^***^ | 0.69^***^ | 0.32^***^ | 0.35^***^ | 0.28^***^ | −0.43^***^ | 0.31^***^ | 0.62^***^ | 0.53^***^ | 0.73^***^ | 0.55^***^ | (0.92) |  |  |  |  | −0.45^***^ |
| PR1 | −0.32^***^ | −0.43^***^ | −0.41^***^ | −0.47^***^ | −0.27^***^ | −0.31^***^ | −0.24^***^ | 0.52^***^ | −0.19^***^ | −0.42^***^ | −0.35^***^ | −0.47^***^ | −0.42^***^ | −0.47^***^ | (0.93) |  |  |  | 0.54^***^ |
| PR2 | −0.34^***^ | −0.35^***^ | −0.39^***^ | −0.3^***^ | −0.28^**^ | −0.21^***^ | −0.22^***^ | 0.36^***^ | −0.12^***^ | −0.25^***^ | −0.18^***^ | −0.33^***^ | −0.22^***^ | −0.24^***^ | 0.43^***^ | (0.93) |  |  | 0.48^***^ |
| PR3 | −.4^***^ | −0.43^***^ | −0.44^***^ | −0.5^***^ | −0.33^***^ | −0.39^***^ | −0.4^***^ | 0.54^***^ | −0.24^***^ | −0.51^***^ | −0.41^***^ | −0.55^***^ | −0.44^***^ | −0.5^**^ | 0.55^***^ | 0.42^***^ | (0.92) |  | 0.63^***^ |
| PR4 | −.39^***^ | −0.52^***^ | −0.49^***^ | −0.54^***^ | −0.37^***^ | −0.37^***^ | −0.32^***^ | 0.58^***^ | −0.23^***^ | −0.48^***^ | −0.38^***^ | −0.57^***^ | −0.46^***^ | −0.53^***^ | 0.58^***^ | 0.46^***^ | 0.75^***^ | (0.93) | 0.66^***^ |
| PR5 | −.43^***^ | −0.44^***^ | −0.49^***^ | −0.46^***^ | −0.33^***^ | −0.33^***^ | −0.33^***^ | 0.54^***^ | −0.21^***^ | −0.45^***^ | −0.35^***^ | −0.52^***^ | −0.52^***^ | −0.45^***^ | 0.54^***^ | 0.48^***^ | 0.63^***^ | 0.66^***^ | (0.92) |
| Opombe.  α^−^= Cronbach α change if item is deleted.  *r_iSc_* = discriminant ceofficient (corrected item total correlation)  Total Cronbach α coefficients (α=.93).  Total reliability coefficients *r_Sj_*=.41  PS8 and PN5 were eliminated from further analysis  **p<.05*. ***p<.01*. ****p<.001.* | | | | | | | | | | | | | | | | | | | |


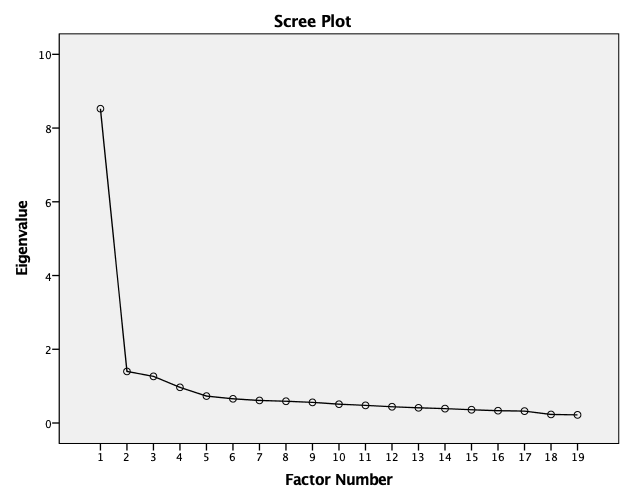


**Figure 2.** Scree Plot for Psychological Functioning at Work Scale

As it can be seen from **Figure 2**, one factor stands out with potential three factor solution, seeming a viable option. From **Table 8**, Eigen values above 1 show that the three−factor solution explains approximately 50.77% of the cumulative variance.

| **Table 8.**  *Maximum Likelihood Initial Eigen Values for Psychological Functioning at Work Scale* | | | | | | | |
| --- | --- | --- | --- | --- | --- | --- | --- |
| Factor | Initial Eigenvalues | | | Extraction Sums of Squared Loadings | | | Rotation Sums of Squared Loadings^a^ |
|  | Total | % of Variance | Cumulative % | Total | % of Variance | Cumulative % | Total |
| 1 | 8.53 | 44.87 | 44.87 | 8.04 | 42.3 | 42.3 | 6.19 |
| 2 | 1.4 | 7.36 | 52.24 | 0.87 | 4.57 | 46.87 | 6.23 |
| 3 | 1.27 | 6.66 | 58.89 | 0.81 | 4.26 | 51.13 | 6.03 |
| 4 | 0.97 | 5.09 | 63.98 |  |  |  |  |
| 5 | 0.73 | 3.85 | 67.83 |  |  |  |  |
| 6 | 0.66 | 3.45 | 71.27 |  |  |  |  |
| 7 | 0.61 | 3.22 | 74.49 |  |  |  |  |
| 8 | 0.59 | 3.11 | 77.6 |  |  |  |  |
| 9 | 0.56 | 2.94 | 80.54 |  |  |  |  |
| 10 | 0.51 | 2.69 | 83.23 |  |  |  |  |
| 11 | 0.48 | 2.52 | 85.74 |  |  |  |  |
| 12 | 0.44 | 2.32 | 88.06 |  |  |  |  |
| 13 | 0.41 | 2.17 | 90.23 |  |  |  |  |
| 14 | 0.39 | 2.05 | 92.28 |  |  |  |  |
| 15 | 0.36 | 1.89 | 94.17 |  |  |  |  |
| 16 | 0.33 | 1.76 | 95.92 |  |  |  |  |
| 17 | 0.32 | 1.7 | 97.62 |  |  |  |  |
| 18 | 0.23 | 1.22 | 98.84 |  |  |  |  |
| 19 | 0.22 | 1.16 | 100 |  |  |  |  |
| *Note.*  Extraction Method: Maximum Likelihood. | | | | | | | |

Three-factor structure shows a good fit, if we look into pattern factor matrix using Keiser Oblimin rotation, which was applied due to high multicollinearity among variables. First factor refers to negative affectivity *("…feel fear”, “…feel concerned”, “... feel sadness.”, “…feel anger”, “…feel concerned”, “…feel exhausted.”*). Second factor encompasses items referring to resilience. All items seem to have a fairly stable factor loadings. Third factor refers to items referring to negative self-regulatory outcomes, these includes items referring to cognitive regulation, as well as emotional regulation, such as the ability to be empathetic or being able to regulate emotions when faced with a conflict situation. One item that doesn’t seem to fit the appropriate factor structure is *“... easily control irritability in a conflict situations.”,* which was added as an item that should have a reversely scored and refers to emotional regulation. Nevertheless, McCann et al. (2013) propose that resilience in healthcare workers also means the ability to have and maintain good relationships at work.

**Table 9.**

*Pattern Matrix for Psychological Functioning at Work scale Maximum Likelihood with Keiser−Oblimin rotation*

|  | Items | Factors | | |
| --- | --- | --- | --- | --- |
|  |  | Negative Affectivity | Resilience | Self-regulatory failures |
| PS1 | ...  have memory problems | −0.06 | −0.06 | **0**.**71** |
| PS2 | ... have difficulties making decisions | 0.09 | −0.11 | **0.62** |
| PS3 | ... have difficulties maintaining attention on work activities you were performing | 0.07 | −0.14 | **0.61** |
| PS4 | ... miss something or not see something important | −0.05 | 0 | **0.68** |
| PS5 | ... in interaction with others said something you later regretted. | 0.14 | −0.06 | **0.45** |
| PS6 | ... make a risky choice | 0.23 | 0.13 | **0.39** |
| PS7 | ... remain indifferent in a situation that required empathy | 0.06 | −0.14 | **0**.**34** |
| PN1 | ... feel fear | **0.59** | −0.12 | 0.15 |
| PN2 | ... feel sadness | **0.67** | −0.1 | 0.05 |
| PN3 | … feel anger | **0**.**58** | −0.02 | 0.09 |
| PN4 | ... feel powerless | **0.69** | −0.17 | 0.06 |
| PN5 | ...feel concerned | **0.85** | −0.06 | −0.04 |
| PN6 | ... feel exhausted | **0.44** | −0.11 | 0.22 |
| PR1 | ... been organised and efficient. | 0.17 | **0.55** | −0.19 |
| PR2 | ... adapt to changes happening around you with ease. | −0.18 | **0.58** | 0.02 |
| PR3 | ... think positively | −0.16 | **0.77** | 0.06 |
| PR4 | ... feel strong and capable when faced with difficulties | −0.13 | **0.81** | 0.04 |
| PR5 | ... have enough energy to handle work tasks | −0.07 | **0.68** | −0.08 |
| PR6 | ... easily control irritability in a conflict situation. | −0.12 | **0.55** | −0.07 |
| *Notes.*  Extraction Method: Maximum Likelihood.  Rotation Method: Oblimin with Kaiser Normalization. | | | | |
|  |  | | | |

**Confirmatory factor analysis**

When performing confirmatory factor analysis, two items on the Psychological Functioning at Work Scale, have shown to be problematic, such as *“…feel exhausted” and “…easily control irritability in a conflict situation”*. Both two items have shown to have high modification indices with items on other dimensions. Which was in line, with theoretical propositions. Based upon that, we have eliminated the two items from the questionnaire and we propose that a new item is added referring to emotional regulation in conflict situation in next version. Maximum Likelihood showed adequate fit to the data for the proposed three-factor structure. Although high *χ^2^* statistics shows not the best fit to the model ( *χ^2^* = 789.96, *p < .001*) one of the problems with *χ^2^*  is that it tends to reject the null hypothesis very quickly in a large sample size. In this case, Schweizer (2010), refers to the *χ^2^/df* statistic when assessing the model fit in large sample sizes, in our case our model is well above the 2-3 ratio, which shows good fit. Nevertheless, the author proposes that in large sample sizes the border can be higher. However, if we refer to *RMSEA*, *CFI*, *TLI* and *SRMR* statistics, our model shows adequate fit to the data (**Table 10**).

**Table 10.**

*Confirmatory factor analysis Maximum Likelihood statistics for different factor models of Psychological Functioning at Work Scale.*

|  | *χ^2^* | *df* | *χ^2^/df* | *p* | *CFI* | *TLI* | *RMSEA* | | *RMSEA* (*90% CI*) | | *p* | *SRMR* |
| --- | --- | --- | --- | --- | --- | --- | --- | --- | --- | --- | --- | --- |
|  |  |  |  |  |  |  |  | *Lower* | | *Upper* |  |  |
| 1 – factor model | 2045.68 | 119 | 24.74 | 0.000 | 0.8 | 0.78 | 0.11 | 0.1 | | .11 | .000 | .07 |
| 2 – factor mode | 1427.66 | 118 | 10.29 | 0.000 | 0.87 | 0.85 | 0.1 | 0.09 | | .1 | .000 | .06 |
| 3 – factor model | 789.96 | 116 | 6.81 | 0.000 | 0.93 | 0.92 | 0.07 | 0.06 | | .07 | .000 | .04 |

If we look at the modification indices, very high modification indices on several items. This is in line with high reliability index and relatively large number of items with medium to high size correlations. Schematically, as well as theoretically several items are similar, such as decision making items, as well as fear and concern item. To improve the model fit we therefore propose to allow eight covariances on items PS2 ~~ PS4, PS7 ~~ PS10, PS3~~PS4, rPS15~~rPS16, PS7 ~~ PS8, PS8 ~~ PS10, PS2~~ PS3 and PS8~~PS14. After these items were allowed to covary. The model showed significant improvement $\chi^{2}=619.02, df=108, p<0.001, \chi^{2}/df=5.73, CFI=0.95, TLI=0.93, RMSEA=0.06, 90\% CI \left( 0.06,0.07 \right), p=0.000,$*SRMR* = 0.04 (**Table 11)**.

**Table 11.**

*Maximum Likelihood Statistics for Psychological Functioning at Work Scale*

| Items |  |  | Reduction χ^2^ |
| --- | --- | --- | --- |
| PS13 | ~~ | r20 | 50.15 |
| PS10 | ~~ | PS11 | 41.28 |
| PS5 | ~~ | PS18 | 37.27 |
| PS7 | ~~ | PS10 | 34.46 |
| PS18 | ~~ | PS19 | 32.2 |
| na | =~ | rPS6 | 31.96 |
| selfregulation | =~ | PS19 | 30.96 |
| selfregulation | =~ | PS5 | 27.42 |
| selfregulation | =~ | PS13 | 24.41 |
| resilience | =~ | PS19 | 24.31 |
| PS3 | ~~ | r16 | 21.72 |
| na | =~ | PS10 | 21.57 |
| PS4 | ~~ | PS10 | 21.26 |
| PS9 | ~~ | PS11 | 19.64 |
| PS3 | ~~ | PS5 | 18.29 |
| PS5 | ~~ | PS19 | 16.8 |
| PS5 | ~~ | PS11 | 16.57 |
| resilience | =~ | PS13 | 16.42 |
| PS9 | ~~ | r15 | 15.72 |
| PS19 | ~~ | rPS6 | 15.53 |
| PS3 | ~~ | r15 | 14.04 |
| P2 | ~~ | PS4 | 12.72 |
| na | =~ | P2 | 12.23 |
| P2 | ~~ | r16 | 12.22 |
| PS13 | ~~ | rPS6 | 11.93 |
| PS10 | ~~ | PS9 | 11.81 |
| P2 | ~~ | PS10 | 11.71 |
| PS3 | ~~ | PS11 | 11.19 |
| PS19 | ~~ | r20 | 11.07 |

**References:**

McCann, C. M., Beddoe, E., McCormik, K. Huggard, P., Kedge, S., Adamson, C., & Huggard, J. (2013). Resilience in the health professionals: A review of recent literature. *International Journal of Wellbeing, 3*(1), 60–81. Doi:10.5502/ijw.v3i1.4

Paul, F., Schredl, M., & Alpers, G. W. (2015). Nightmares affect the experience of sleep quality but not sleep architecture: an ambulatory polysomnographic study.*Borderline Personality Disorder and Emotion Dysregulation, 2* (3), 1–9.

Schweizer, K. (2010). Some guidelines concerning the modelling of traits and abilities in test construction. *Eur. J. Psychol. Assess.* 26, 1–2. doi: 10.1027/1015- 5759/a000001
